# Optimization of the fluorogen-activating protein tag for quantitative protein trafficking and colocalization studies in *S. cerevisiae*

**DOI:** 10.1091/mbc.E24-04-0174

**Published:** 2024-07-01

**Authors:** Katherine G. Oppenheimer, Natalie A. Hager, Ceara K. McAtee, Elif Filiztekin, Chaowei Shang, Justina A. Warnick, Marcel P. Bruchez, Jeffrey L. Brodsky, Derek C. Prosser, Adam V. Kwiatkowski, Allyson F. O’Donnell

**Affiliations:** a Department of Biological Sciences, University of Pittsburgh, PA 15260; b Molecular Biosensor and Imaging Center, Carnegie Mellon University, Pittsburgh, PA 15213; c Department of Biology, Virginia Commonwealth University, Richmond, VA 23284; d Department of Cell Biology, School of Medicine, University of Pittsburgh, Pittsburgh, PA 15261; MRC Laboratory of Molecular Biology

## Abstract

Spatial and temporal tracking of fluorescent proteins (FPs) in live cells permits visualization of proteome remodeling in response to extracellular cues. Historically, protein dynamics during trafficking have been visualized using constitutively active FPs fused to proteins of interest. While powerful, such FPs label all cellular pools of a protein, potentially masking the dynamics of select subpopulations. To help study protein subpopulations, bioconjugate tags, including the fluorogen activation proteins (FAPs), were developed. FAPs are comprised of two components: a single-chain antibody (SCA) fused to the protein of interest and a malachite-green (MG) derivative, which fluoresces only when bound to the SCA. Importantly, the MG derivatives can be either cell-permeant or -impermeant, thus permitting isolated detection of SCA-tagged proteins at the cell surface and facilitating quantitative endocytic measures. To expand FAP use in yeast, we optimized the SCA for yeast expression, created FAP-tagging plasmids, and generated FAP-tagged organelle markers. To demonstrate FAP efficacy, we coupled the SCA to the yeast G-protein coupled receptor Ste3. We measured Ste3 endocytic dynamics in response to pheromone and characterized *cis*- and *trans*-acting regulators of Ste3. Our work significantly expands FAP technology for varied applications in *S. cerevisiae*.

## Abstract (executive summary)

Quantitative endocytic assays are required to characterize factors that regulate both ligand-dependent and constitutive endocytosis.

We optimize the fluorogen-activating protein (FAP) technology for use as a live cell imaging probe in yeast that fluoresces in the far-red range for quantitative endocytosis assays.

The FAP tools and approaches generated will facilitate quantitative endocytic and protein recycling assays for yeast cell biologists.

## INTRODUCTION

Eukaryotic cells control the distribution of proteins within membrane-bound organelles via selective protein trafficking. At the plasma membrane (PM), protein abundance is regulated by both exocytic and endocytic events. Achieving the correct balance of PM proteins is critical for nutrient homeostasis and dynamic cellular responses, such as adaptation to stress, hormones, or other signaling molecules (Sorkin and Von Zastrow, 2002; O’Donnell and Schmidt, 2019; Hager *et al.*, 2021). Defective membrane trafficking or aberrant activity of membrane proteins at the PM cause disease, including diabetes, cardiac arrhythmias, and Alzheimer’s (Aridor and Hannan, 2000; Howell *et al.*, 2006; Hung and Link, 2011; Yarwood *et al.*, 2020). To better define how defective protein trafficking contributes to these and other disorders, it is necessary to first distinguish changes in protein activity from alterations in protein localization (Perkins and Bruchez, 2020). For example, mutations affecting the synthesis, folding, trafficking, or activity (i.e., gating or conductance) of the cystic fibrosis transmembrane conductance regulator (CFTR) all cause cystic fibrosis. Still, unique therapies that improve CFTR function are only effectively deployed when the underlying molecular mechanism for disease is understood (Moskowitz *et al.*, 2008; Koulov *et al.*, 2010; Coppinger *et al.*, 2012; Lopes-Pacheco, 2016, 2019). Quantitative studies of endocytosis and intracellular protein sorting are imperative to identify and characterize mutations that compromise protein trafficking.

Techniques such as cell-surface biotinylation assays and ligand labeling are frequently used to quantify protein trafficking to and from the PM, yet both have their limitations (Nishimura and Sasaki, 2008; Bohme and Beck-Sickinger, 2009; Chen *et al.*, 2011; Tham and Moukhles, 2017). For example, cell-surface biotinylation can abnormally trigger endocytosis (Dundas *et al.*, 2013) and may underestimate the abundance of single-pass membrane proteins (Tham and Moukhles, 2017). In addition, biotinylation of surface proteins is ineffective in some cell types and model systems. For example, in yeasts like *Saccharomyces cerevisiae* and *Candida albicans*, avidin nonspecifically binds the cell wall (Masuoka *et al.*, 2002), while in select human cell types, some membrane proteins associate nonspecifically with streptavidin (Cole *et al.*, 1987). Furthermore, extracting biotinylated membrane proteins can be problematic; in yeast and plants, the cell wall provides an added barrier to effective membrane protein solubilization and isolation (Smith *et al.*, 2000; Klis *et al.*, 2002; Powell *et al.*, 2003; Mukherjee *et al.*, 2020). Likewise, quantitative endocytic assays using receptor-specific ligand probes also have limitations and require selective derivatization of each ligand. The modified ligands must also be PM-impermeant, bind the receptor similar to native ligand, and remain bound to monitor internalization and postendocytic sorting (Los *et al.*, 2008; Komatsu *et al.*, 2011; Leng *et al.*, 2017; Jonker *et al.*, 2020). Radiolabeled or rhodamine-labeled epidermal growth factor (EGF) has been a powerful tool for studying the endocytosis, sorting, and recycling of the epidermal growth factor receptor (EGFR) in many cell types and models (Huang *et al.*, 2007; Goh *et al.*, 2010; Tomas *et al.*, 2014; Tanaka *et al.*, 2018). Similarly, in *S. cerevisiae*, radiolabeled α-factor, the mating pheromone that binds the G-protein-coupled receptor (GPCR) Ste2, was initially used to study Ste2 endocytic dynamics (Zanolari and Riezman, 1991; Hicke *et al.*, 1998; Dunn and Hicke, 2001; Shih *et al.*, 2002; Toshima *et al.*, 2009). Later, fluorescently-tagged α-factor was used to stimulate Ste2 internalization and illuminate its ligand-induced trafficking (Toshima *et al.*, 2006). Although useful, these tools are not easily adapted to explore the many proteins trafficked to and from the PM.

In addition to cell-surface biotinylation and ligand labeling, fluorescent proteins (FPs) such as the green fluorescent protein (GFP) and its derivatives are widely used to monitor membrane protein dynamics (Lippincott-Schwartz *et al.*, 2003; Miyawaki *et al.*, 2003). Despite the wealth of applications for FPs and the bounty of available probes, FPs have some shortcomings. For example, high fluorescence levels in intracellular compartments can sometimes prevent clear differentiation of the PM pool of a tagged protein. This intracellular fluorescence can complicate quantitative measures of endocytic turnover or PM delivery. While the use of pH-sensitive FP derivatives, such as the GFP derivative pHluorin, facilitates quantitative endocytic assays by quenching the fluorescence of intracellular subpopulations (Balaji and Ryan, 2007; Prosser *et al.*, 2010; Nicholson-Fish *et al.*, 2016; Prosser *et al.*, 2016), this approach requires transit to an acidic organelle, for example, the multivesicular body (MVB) or lysosome (yeast vacuole equivalent). Furthermore, these pH-sensitive probes are ineffective in recycling assays that assess the return of endocytosed membrane proteins to the PM.

As an alternative to FPs, bioconjugate tags can permit temporal and spatial fluorescent labeling and simultaneous visualization of specific proteins. Unlike FPs, some bioconjugate tags form covalent bonds with chemical fluorophores (Griffin *et al.*, 1998); thus, light is emitted when a peptide tag binds the probe. However, these probes exhibit background fluorescence due to their affinity for cysteine-rich proteins (Griffin *et al.*, 1998; Zurn *et al.*, 2010; Gallo, 2020). In contrast, appended Halo, CLIP, SNAP, LAP, and BL motifs engineered onto proteins of interest form irreversible bonds with fluorescently labeled ligands (Keppler *et al.*, 2003; Gautier *et al.*, 2008; Los *et al.*, 2008; Watanabe *et al.*, 2010; Yao *et al.*, 2012). These tags then fluoresce when bound to an added dye, which has advantages for spatial and temporal resolution (Griffin *et al.*, 1998; Keppler *et al.*, 2003; Keppler *et al.*, 2004; Chen *et al.*, 2005; Fernandez-Suarez *et al.*, 2007; Luedtke *et al.*, 2007; Gautier *et al.*, 2008; Gallo, 2020).

**FIGURE 1:**
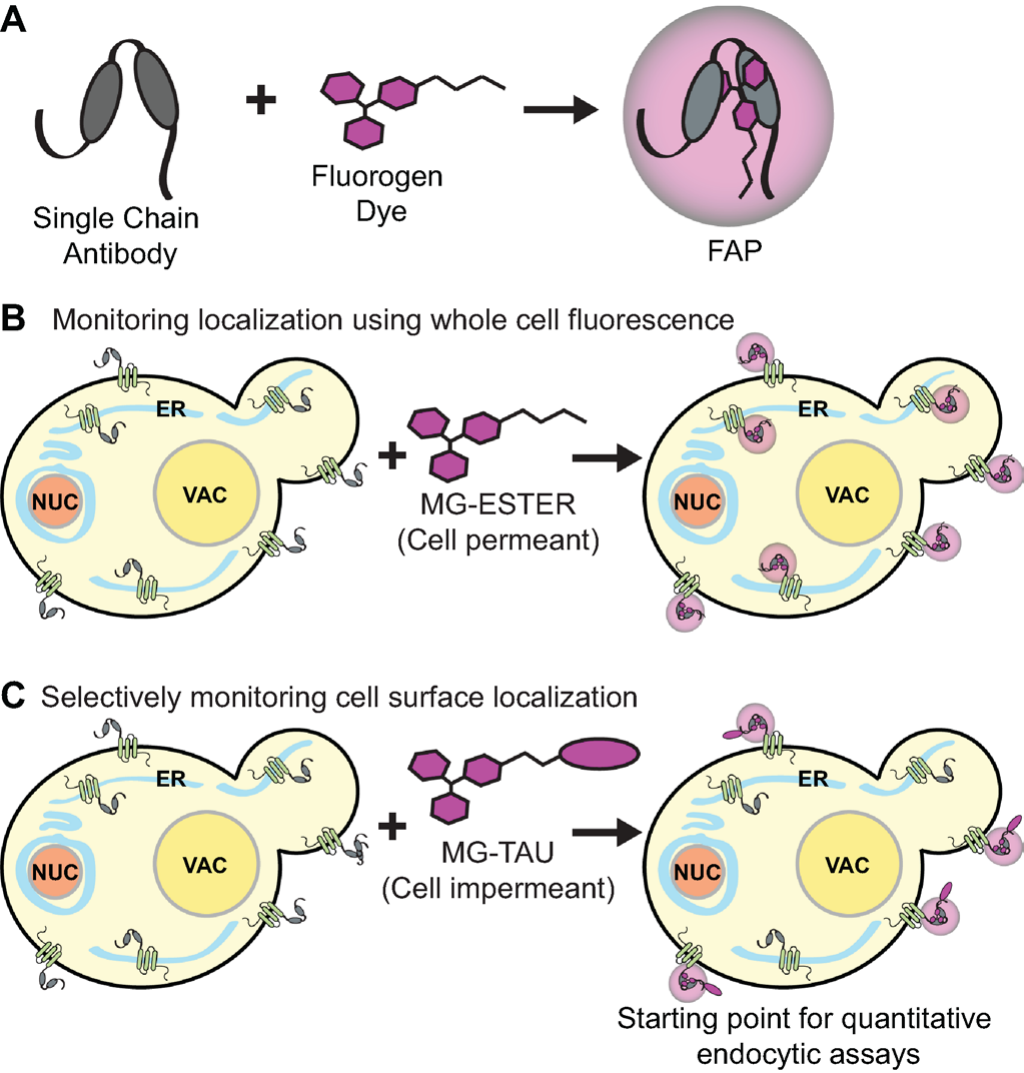
Model of FAP technology. (A) A SCA binds the fluorogen dye, locking it into a conformation that promotes fluorescence. (B and C) The fluorogen dye can be cell-permeant (*i.e*., MG-ESTER) or -impermeant (*i.e*., MG-TAU). While MG-ESTER allows for visualization of the entire pool of SCA-tagged protein, MG-TAU allows for selective visualization of the cell surface pool. By adding dye for a brief time and washing excess away, one can quantitively monitor endocytosis of the PM-labeled pool from the cell surface.

Another system for bioconjugate tagging is the use of fluorogen-activating protein (FAP) technology, which offers selective labeling of specific membrane protein pools, enhanced spatiotemporal visualization, and added flexibility in imaging parameters and approaches (Szent-Gyorgyi *et al.*, 2008; Boeck and Spencer, 2017; Emmerstorfer-Augustin *et al.*, 2018; Hager *et al.*, 2018; Gallo, 2020; Perkins and Bruchez, 2020). FAP tags are comprised of a genetically encoded single-chain antibody (SCA) fused to a protein of interest. The variable region of the SCA displays a high affinity for synthetic compounds, that is, fluorogens. When free, neither the SCA nor the fluorogen are fluorescent (Figure 1; Szent-Gyorgyi *et al.*, 2008; Fisher *et al.*, 2010; Gallo, 2020; Perkins and Bruchez, 2020). However, when the fluorogen binds the SCA, the fluorogen conformation is restricted, resulting in a significant (up to 20,000-fold) increase in fluorescence (Lee *et al.*, 1986; Silva *et al.*, 2007; Shank *et al.*, 2013; Gallo, 2020), thus matching the sensitivity and intensity of conventional FPs such as EGFP and mCherry (Szent-Gyorgyi *et al.*, 2013). Based on this exceptional increase in fluorescence, FAPs also offer a better signal-to-noise ratio than FPs, and the dye does not need to be washed away for certain applications (Perkins and Bruchez, 2020). FAP emission wavelengths are also wide-ranging and adaptable to a variety of filter sets, because the fluorogens can be derived from several dyes, including malachite green (MG). Another key feature of FAP technology is the ability to use either cell-permeant or -impermeant fluorogens (Figure 1, B and C). In mammalian cells, FAPs have been used to examine endocytosis, sorting, and recycling of GABA_A_ receptors in neurons (Lorenz-Guertin *et al.*, 2017), endocytosis of β_2_-adrenergic receptors (β_2_AR) in NIH3T3 fibroblasts (Holleran *et al.*, 2010), β_2_AR shuttling to and from the PM in lymphoma cells (Fisher *et al.*, 2010), and mutant CFTR trafficking to the PM in human bronchial epithelial cells and HEK293T cells (Holleran *et al.*, 2012; Goeckeler-Fried *et al.*, 2021). Further, permeant fluorogens can be used in conjunction with impermeant fluorogens to reveal total cellular protein versus the cell surface pool, shedding light on changes in protein turnover and trafficking to a specific organelle (Pratt *et al.*, 2015; Yan *et al.*, 2015; Naganbabu *et al.*, 2016; Perkins and Bruchez, 2020). FAP technology can also be adapted to measure PM recycling rates when two differentially fluorescent fluorogens are used (Szent-Gyorgyi *et al.*, 2013; Shiwarski *et al.*, 2017).

Despite its extensive uses, the FAP technology has not been widely adapted for use in the budding yeast *S. cerevisiae* even though SCAs were developed as a result of yeast screens (Feldhaus *et al.*, 2003; Szent-Gyorgyi *et al.*, 2008). Nevertheless, we and others have recently used FAP technology to successfully track the sorting of membrane proteins (Emmerstorfer-Augustin *et al.*, 2018; Hager *et al.*, 2018). Here, we significantly expand on these initial studies by enhancing the performance and accessibility of FAP technology in yeast. We codon-optimized the SCA for expression in *S. cerevisiae* and confirmed that optimized FAP exhibits increased protein stability. We then created an extensive collection of FAP-tagged cloning vectors and built a suite of FAP-tagged cellular markers for colocalization studies in yeast. We demonstrated the utility of the FAP-tagging approach by characterizing *cis*- and *trans*-acting regulators of the ligand-induced endocytosis of Ste3, the GPCR that controls mating pathway signaling in Mat α yeast cells (Bardwell *et al.*, 1994; Bardwell, 2005). We expect these new FAP tools to be a rich, widely applicable resource for the yeast cell biology community. FAP-tagged Ste3 and other membrane cargos will also allow us to define the factors needed for postendocytic recycling in future studies.

## RESULTS

### FAP codon optimization for yeast expression

**FIGURE 2:**
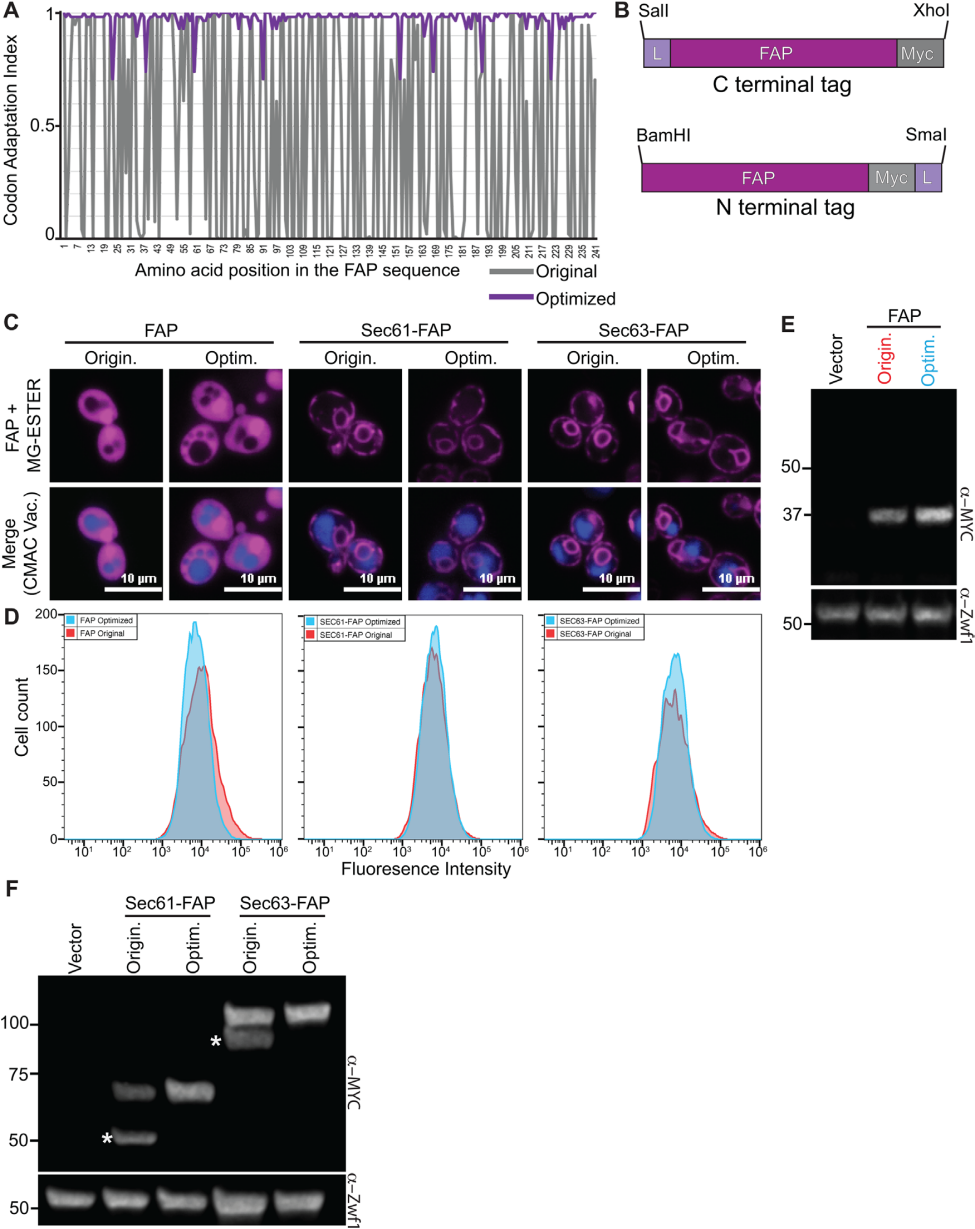
Codon optimization of the FAP sequence for expression in yeast. (A) Graph of the codon adaptation index for *S. cerevisiae* of the original FAP sequence (grey) versus the codon-optimized FAP sequence (purple). (B) Schematic of the N- or C-terminal FAP tagging cassettes. L = linker (Ala-Gly)_3_ and Myc = 2xMYC epitope tag. (C) Confocal fluorescence microscopy of yeast expressing soluble FAP, Sec61- or Sec63-FAP, either as the original (Origin.) or the codon-optimized FAP sequences (Optim.; magenta). Vacuoles are stained with CMAC (blue) in the merge. (D) Histograms of flow cytometry data showing fluorescence distributions for cells expressing the different FAP-fusions from panel C. (E and F) Immunoblots of yeast extracts from cells expressing an empty vector control, and either (E) soluble FAP or (F) FAP-tagged Sec61 or Sec63 as original or optimized versions. Zwf1 is the loading control. The white asterisks in (F) identify breakdown products observed for the FAP_Origin_ tagged proteins. Numbers on the left represent MW markers in kDa.

We first codon optimized the SCA motif (described as dL5 in [Szent-Gyorgyi *et al.*, 2008; Yan *et al.*, 2015]) for expression in *S. cerevisiae* using the JAVA Codon Adaptation Tool (JCat; Grote *et al.*, 2005). In JCat, the Codon Adaption Index (CAI) ranges from zero to one, where values approaching one indicate a codon with an abundant tRNA in that organism. Before optimization, the original FAP (FAP_origin_) had an average CAI of 0.063, indicating that most codons were matched to low abundance yeast tRNAs. In contrast, the codon-optimized FAP (FAP_optim_) sequence had a CAI value of 0.973 (Figure 2A). We also introduced an N- or C-terminal linker (sequence Ala-Gly-Ala-Gly-Ala-Gly) to facilitate FAP folding independently of the protein to which it was fused, and a MYC epitope to facilitate biochemical detection (Figure 2B).

We then sought to determine whether the FAP_optim_ improved expression and function in yeast. Both FAP_optim_ and FAP_origin_ were expressed under the control of the *TEF1* promoter as a soluble protein (“FAP”) or as fusions to the ER-resident membrane proteins Sec61 and Sec63. Using flow cytometry and fluorescence micro­scopy, we found no difference in the fluorescence intensity of FAP_optim_ compared with FAP_origin_ regardless of whether they were expressed as free soluble or fusion proteins (Figure 2, C and D; Supplemental Figure 1, A and B). While free FAP_origin_ and FAP_optim_ were roughly equivalent on immunoblots, when the FAP-tagged fusion proteins were examined via immunoblotting, we consistently observed breakdown products with FAP_origin_-fused proteins (Figure 2, E and F). For Sec61- and Sec63-FAP_origin_, we observe bands at the expected molecular masses for intact fusions (75 and 105 kDa, respectively), but significant breakdown products were also detected (∼50 and 90 kDa, respectively; Figure 2F). Because the MYC tag detected in these Sec61- and Sec63-FAP_origin_ fusions is at the C-terminus of the protein, these breakdown products must be due to cleavage within the Sec61 or Sec63 proteins themselves and not in the FAP tag, which is only 24.2 kDa. Importantly, these breakdown products were absent with Sec61- and Sec63-FAP_optim_ fusions (Figure 2F), suggesting that proteins tagged with FAP_origin_ are more susceptible to cleavage and degradation. If present in live cells, these degradation products could lead to spurious determinations of protein localization. Therefore, all subsequent studies in our work used the FAP_optim_ moiety.

### Cellular growth conditions and vacuolar proteases influence FAP fluorescence

**FIGURE 3:**
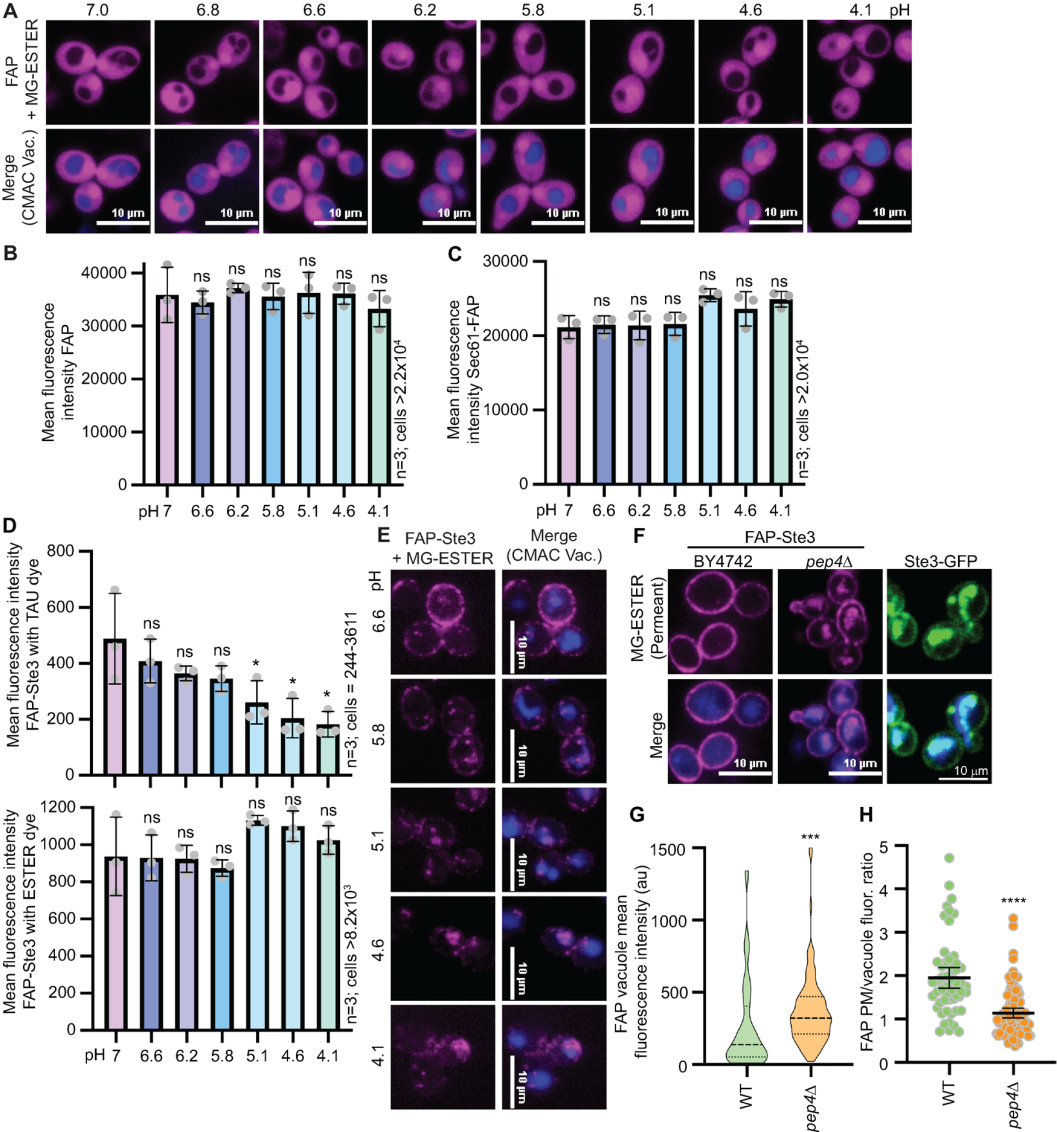
Parameters that impact FAP fluorescence in yeast. (A and E) Confocal fluorescence microscopy images of cells expressing (A) soluble FAP_OPTIM_ or (E) FAP_OPTIM_-Ste3. Cells were incubated in different pH media for 2 h before adding MG-ESTER (magenta) and CMAC (blue) dyes and imaging. (B and C) Bar graphs representing the mean of three biological replicates of flow cytometry data for cells from in panel A (in panel B) or those expressing FAP-Sec61 (in panel C). ANOVA statistical test assessed changes in fluorescence between samples relative to pH 7.0 (ns = not significant). (D) Bar graphs of the mean FAP-Ste3 fluorescence intensities with MG-TAU (top) or -ESTER (bottom) dyes from three replicate flow cytometry experiments are shown. ANOVA statistical test assessed changes in fluorescence between samples relative to pH 7.0 (ns = not significant; * *p* < 0.05). (F) Confocal fluorescence microscopy of cells expressing either FAP-Ste3 (magenta) or Ste3-GFP (green) in the indicated genetic backgrounds. (G and H) Quantification of the vacuole signal intensity (violin plot) or the PM/vacuole fluorescence intensity ratio (dot plot) for the FAP images in panel F. Student’s *t* test defines a statistically significant *p* value < 0.0005 in (G) and *p* value < 0.00005 in (H) for the WT to *pep4*∆ comparison.

Next, we explored conditions that might impact FAP_optim_ signal intensity. The FAP_origin_ signal is reportedly influenced by changes in pH, with reduced fluorescence at the cell surface of the Ste2 receptor in acidic environments and an optimal fluorescent signal at pH 6.5 (Emmerstorfer-Augustin *et al.*, 2018). We found that soluble, intracellular FAP_optim_ and Sec61-FAP_optim_ were unaffected by pH changes, with equivalent fluorescence intensities observed in cells incubated at pHs ranging from 4.1 to 7.0 by both confocal microscopy and flow cytometry (Figure 3, A–C; Supplemental Figure 2, A–E). These data indicate that intracellular FAP_optim_ probes can be used in yeast cells grown in media at a wide range of pHs. This may be partly due to the fact that even yeast cells grown in acidic conditions (∼pH 4.0) can buffer their cytosols to maintain a near-optimal intracellular pH of 6 (Valli *et al.*, 2005).

While intracellular FAP-tagged probes were unaffected by the pH of the growth medium, we wondered whether a cell-surface membrane protein would behave similarly. We expressed FAP-tagged Ste3 with an extracellular N-terminal FAP_optim_ tag directly exposed to environmental pH changes and quantitatively monitored its fluorescence by flow cytometry and microscopy. We observed no change in total fluorescence intensity in cells grown in a range of pHs when the fluorogen was activated using the cell permeant MG-ESTER dye. However, there was a dramatic increase in receptor internalization with decreasing pH (Figure 3E), with little-to-no receptor detected at the PM in cells that were shifted into acidic medium (pH 4.1–5.1). Notably, an acidic pH-induced increase in endocytosis has been reported previously in yeast (Motizuki *et al.*, 2008). We speculate that the low pH used here caused a similar increase in endocytosis given the observed shift in FAP_optim_-Ste3 localization from the PM to intracellular compartments as the pH was reduced (Figure 3E). This pH-dependent relocalization may also explain earlier observations with FAP-Ste2 (Emmerstorfer-Augustin *et al.*, 2018).

In contrast to the MG-ESTER results, we observed an ∼twofold reduction in fluorescence at pH 4.1 compared to pH 7.0 with the cell impermeant MG-TAU dye (Figure 3, D and E; Supplemental Figure 2, F and G). In addition, there was a dramatic decrease in the number of cells with sufficient fluorescent signal to reach the gating threshold in flow cytometry experiments with MG-TAU at low pH (Supplemental Figure 2, F and G; <20% of cells were fluorescent). In contrast, there was no change in the percentage of cells measured across the pH ranges with the permeant MG-ESTER derivative (Supplemental Figure 2, F and G). While these results might be due to pH sensitivity of the FAP/MG-TAU probe combination, given the shift in distribution of Ste3 observed with the MG-ESTER dye (Figure 3E), it more likely reflects the loss of cell surface Ste3 due to acidic pH-induced endocytosis.

During these initial experiments, we noted little FAP-tagged Ste3 vacuolar fluorescence under steady-state conditions or when the protein was internalized in response to acidic pH stress (Figure 3E). Instead, we observed several puncta peripheral to the vacuole, likely prevacuolar endosomes (Figure 3, E and F). This result was unexpected because there is substantial Ste3 sorting to the vacuole under steady-state conditions, resulting in bright vacuolar fluorescence for receptors tagged with GFP or mCherry (Figure 3F, see Ste3-GFP; Urbanowski and Piper, 2001; Toshima *et al.*, 2014; MacDonald and Piper, 2017; MacDonald *et al.*, 2020). The pH of the yeast vacuole is ∼6 (Kane, 2006), and should be compatible with robust FAP fluorescence in this compartment. However, a key feature of both GFP and mCherry is a stable beta-barrel secondary structure that is recalcitrant to digestion by vacuolar proteases and thus allows fluorescence to persist in vacuoles (Li *et al.*, 1999; Nikko and Pelham, 2009).

We hypothesized that the FAP-containing SCA motif is either protease-sensitive or that FAP-tagged proteins are poorly trafficked to the vacuole. To differentiate between these scenarios, we examined FAP-Ste3 fluorescence in wildtype (WT, BY4742) cells and cells deleted for the master protease Pep4, which lacks >90% of vacuolar protease activity (Woolford *et al.*, 1986). Strikingly, we found that vacuolar FAP fluorescence significantly rose in *pep4*∆ cells compared with WT yeast, thereby decreasing the PM/vacuole fluorescence ratio (Figure 3, F–H). Thus, the loss of vacuolar fluorescence for the FAP-tagged protein in WT cells is due to the susceptibility of the FAP tag to proteolytic digestion in the yeast vacuole and not defective trafficking of FAP-tagged proteins to the vacuole. Notably, loss of the vacuolar signal for FAP-tagged proteins is advantageous as it facilitates protein detection in other cellular locations that might otherwise be masked by bright vacuolar fluorescence, as occurs with GFP and mCherry. Thus, FAP exhibits similar properties as pHluorin, a variant of GFP that loses fluorescence in the acidic environment of the vacuole (Prosser *et al.*, 2010; Prosser *et al.*, 2016), but with the added adaptability of visualizing vacuolar fluorescence if needed by deleting or inhibiting vacuolar proteases (i.e., *pep4*∆ or pepstatin treatment; Umezawa *et al.*, 1970; Woolford *et al.*, 1986).

### Construction of FAP _optim_ tagging vectors for yeast expression and protein colocalization studies

**FIGURE 4:**
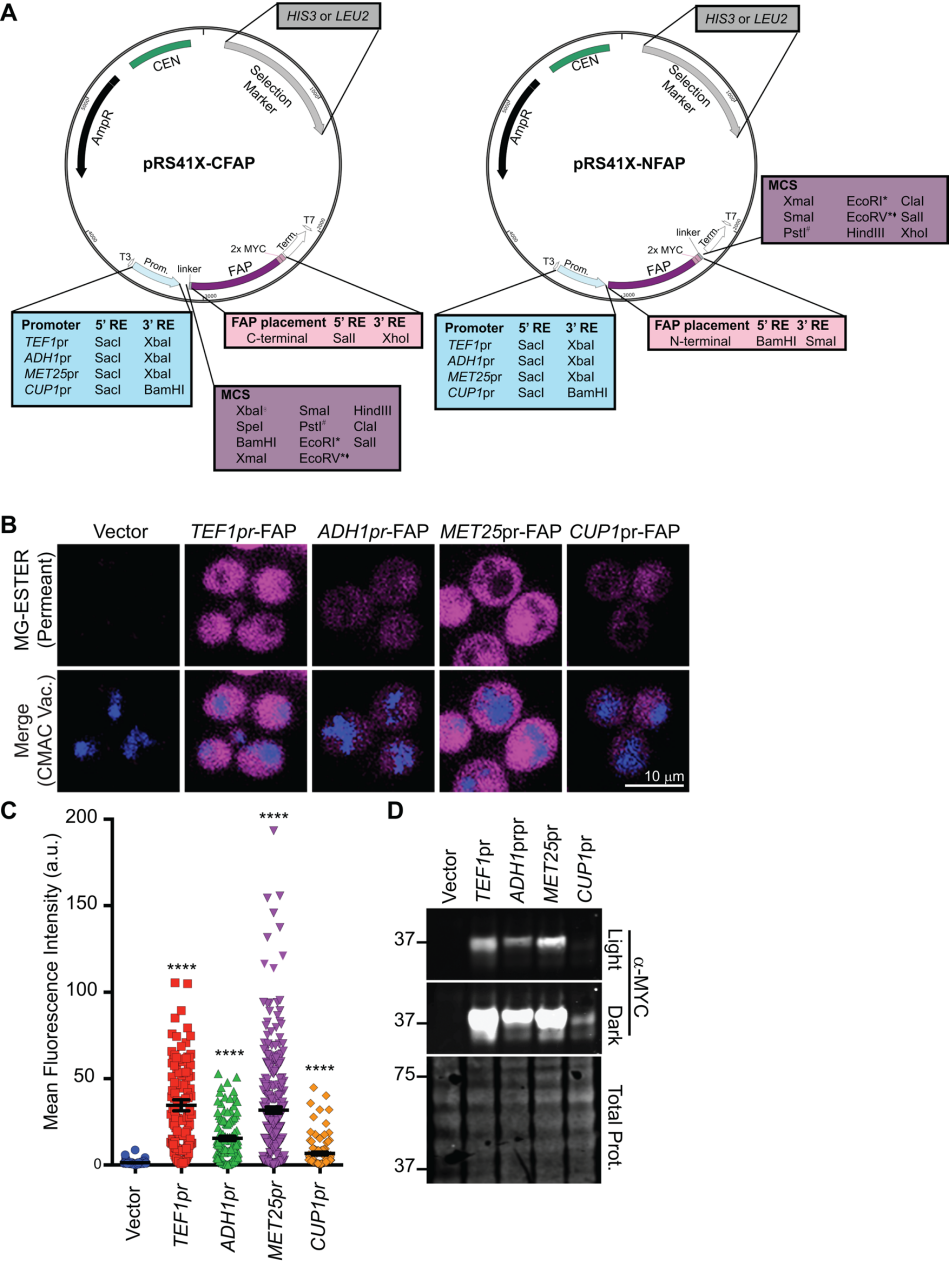
FAP-tagging vectors for use in yeast. (A) Plasmid maps of the cloning vectors built to express N- or C-terminal fusions of FAP_OPTIM_ from the promoters indicated are shown. Plasmids contain either *HIS3* or *LEU2* for selection, and the promoters and FAP sequence positions indicated. (B) Confocal fluorescence microscopy of soluble FAP_OPTIM_ (magenta) expressed in WT cells from plasmids containing the indicated promoters. For *MET25pr* and *CUP1pr*, images are captured at 2 h post induction. CMAC (blue) was used to visualize vacuoles. (C) Mean whole cell fluorescence intensity of FAP_OPTIM_ signal from confocal microscopy from panel B. Kruskal-Wallis with Dunn’s post hoc test was performed, and statistical comparisons are made relative to the vector only control (*p* < 0.0005 = ***). (D) Immunoblot of whole cell extracts from WT cells expressing the FAP_OPTIM_ from the indicated promoter. For *MET25pr* and *CUP1pr*, the extracts were made from cells at 2 h postinduction. A full time-course of induction for these promoters is shown in Supplemental Figure S3. MW markers on the left are in kilodaltons.

To provide a more widely applicable resource for the yeast cell biology community, we generated a collection of FAP_optim_-tagging vectors in which FAP-fusion protein expression can be driven from a range of promoters, each with distinct regulatory features (Figure 4A). The plasmids are based on the commonly used pRS415 or pRS413 vectors (Sikorski and Hieter, 1989; Christianson *et al.*, 1992; Mumberg *et al.*, 1995) that contain the *LEU2* or *HIS3* selectable markers, respectively. FAP_optim_-fusion protein expression can be optimized by incorporating the following promoters: i) *TEF1pr*, for high-level constitutive expression (Mumberg *et al.*, 1995); ii) *ADH1pr*, for modest levels of constitutive expression (Mumberg *et al.*, 1995); iii) *CUP1pr*, for copper-inducible expression (Labbe and Thiele, 1999); and iv) *MET25pr*, for tunable methionine-repressible expression (Kerjan *et al.*, 1986; Figure 4A). We also cloned the FAP_optim_-MYC sequence into two distinct multiple cloning site locations: i) between the *Xho*I and *Sal*I restriction sites for proteins that will be tagged at their C-terminus with FAP_optim_ or ii) between the *BamH*I and *Sma*I restriction sites for proteins that will be N-terminally tagged with FAP_optim_ (Figure 4A).

To assess FAP expression and ensure the promoters functioned as expected, FAP_optim_ was expressed as a soluble protein from each of the promoters in WT cells. The constitutive promoter *TEF1* and the repressible *MET25* promoter (6 h after methionine removal) yielded similar FAP-fluorescence intensities and protein expression (Figure 4, B–D). As expected, the *ADH1pr* and *CUP1pr* (120 min post copper-induction) produced significantly lower levels of FAP than the *TEF1* or *MET25* promoters (Figure 4, B–D; Mumberg *et al.*, 1995; Labbe and Thiele, 1999). An expanded time course of copper induction or methionine repression with the *CUP1* and *MET25* promoters, respectively, to determine the optimal induction timing for FAP_optim_, is shown in Supplemental Figure 3, A–F. We also inserted an ER-targeting sequence upstream of the N-terminal FAP_optim_ tag to facilitate protein entry into the secretory pathway (Supplemental Figure S4A). Finally, we modified constructs to facilitate chromosomal integration of the FAP tag (Supplemental Figure S4B). In sum, we generated 17 new cloning vectors for expressing or tagging FAP proteins (Supplemental Table S2) to maximize the system’s versatility.

**FIGURE 5:**
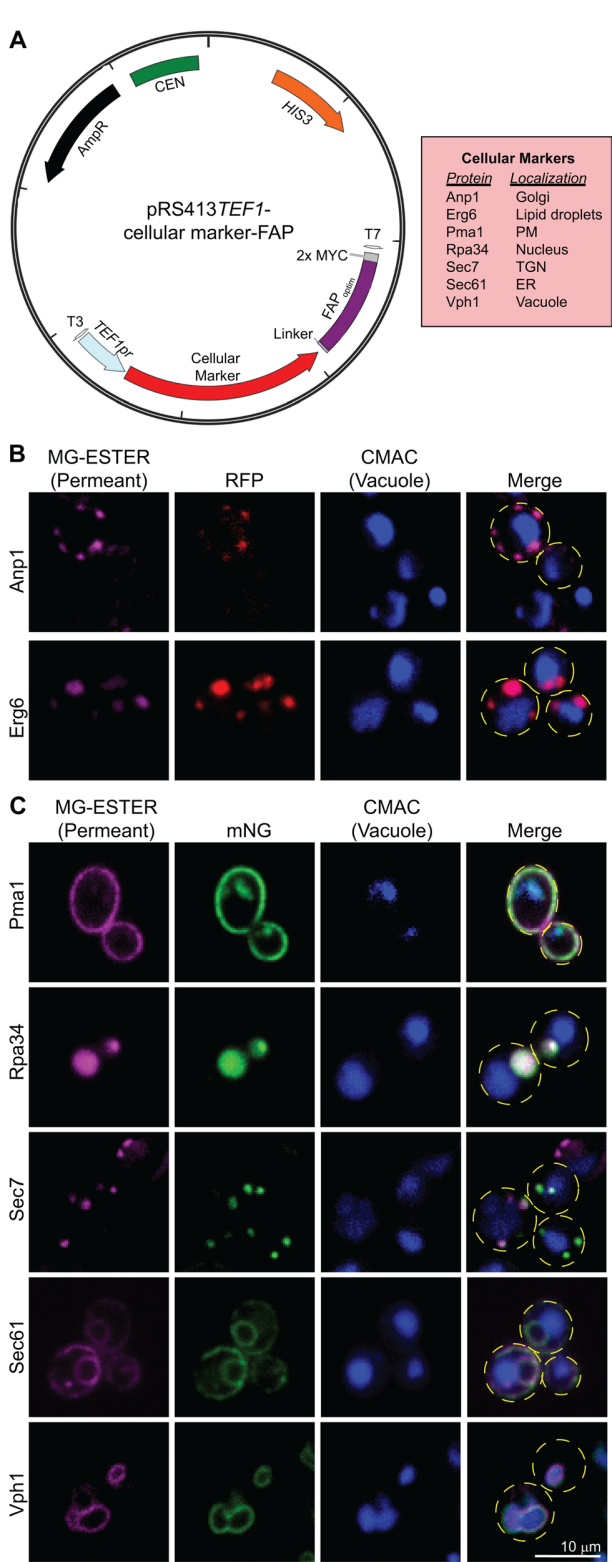
FAP-tagged cellular markers. (A) Schematic of plasmids expressing C-terminal fusions to FAP_OPTIM_ for the genes indicated in the red box at right. (B and C) Confocal fluorescence microscopy of WT cells expressing the FAP_OPTIM_-tagged proteins indicated (magenta) as well as the same protein RFP-tagged (panel B, red) or mNG-tagged (panel C, green). Cell outlines are shown as yellow dashed lines in the merge for reference, and CMAC (blue) stains the vacuoles.

To further aid in the implementation of FAP technology in yeast research, we created a suite of cellular proteins tagged with FAP_optim_ for use in colocalization studies (Supplemental Table S2). These probes were cloned into the pRS413-*TEF1pr* plasmid for constitutive expression (Figure 5A; Supplemental Table S2). We created FAP_optim_-tagged markers for the *cis*-Golgi (Anp1), lipid droplets (Erg6), the PM (Pma1), the nucleus (Rpa34), the *trans*-Golgi (Sec7), the ER (Sec61 and Sec63), and the vacuolar membrane (Vph1; Deshaies and Schekman, 1987; Kane, 1999; Todorow *et al.*, 2000; Young *et al.*, 2001; Glick and Nakano, 2009; Albert *et al.*, 2011; Young *et al.*, 2024). To ensure correct localization, markers were coexpressed in cells in which the respective endogenous protein was tagged with either RFP (Huh *et al.*, 2003) or mNeonGreen (mNG; Meurer *et al.*, 2018), and colocalization was assessed by live cell fluorescence microscopy in the presence of MG-ESTER (Figure 5, B and C). For all the organelle markers examined, we confirmed that the FP- and FAP-tagged proteins colocalized at the expected locations in the cell (Figure 5, B and C). While these findings show that the FAP- and FP-tagged organelle markers behave similarly, it should be noted that we did not functionally assess these FAP-tagged probes (i.e., functional complementation assays have not been done for all FAP-tagged organelle markers).

### FAP _optim_-tagged Ste3 is a functional a-factor receptor

We next sought to use the FAP_optim_ system to define the contributions of *cis*- and *trans*-acting regulators of ligand-induced Ste3 trafficking. The dynamics of Ste3 post pheromone (a-factor) addition have not yet been characterized by live cell imaging as previous studies relied predominantly on biochemical assays to define the Ste3 trafficking itinerary (Zanolari *et al.*, 1992; Chen and Davis, 2000, 2002). These past studies identified regulatory sequences within the C-tail of Ste3 that control its internalization. Transacting regulators have also been described, mainly for the constitutive endocytosis and recycling of Ste3 (Chen and Davis, 2002; Prosser *et al.*, 2015; MacDonald and Piper, 2017; Laidlaw *et al.*, 2022, a, b). Here, we use FAP-tagged Ste3 to assess the role of these regulators in ligand-induced endocytosis of the receptor. Previously, FP-based studies of Ste3 endocytic dynamics were thwarted by the fact that bright fluorescent signal accumulates in the vacuoles from FP-tagged Ste3 (Urbanowski and Piper, 2001; MacDonald and Piper, 2017), which occluded detection of tagged Ste3 at other intracellular locales. In addition, Ste3 gene expression is dramatically increased post a-factor addition (Sprague *et al.*, 1983), muddying isolated analysis of the pre- and postligand expressed pools. However, FAP-Ste3 allows us to fluorescently label a single pool of this receptor at the PM and monitor its localization over time in a fluorescence-based “pulse-chase” assay.

**FIGURE 6:**
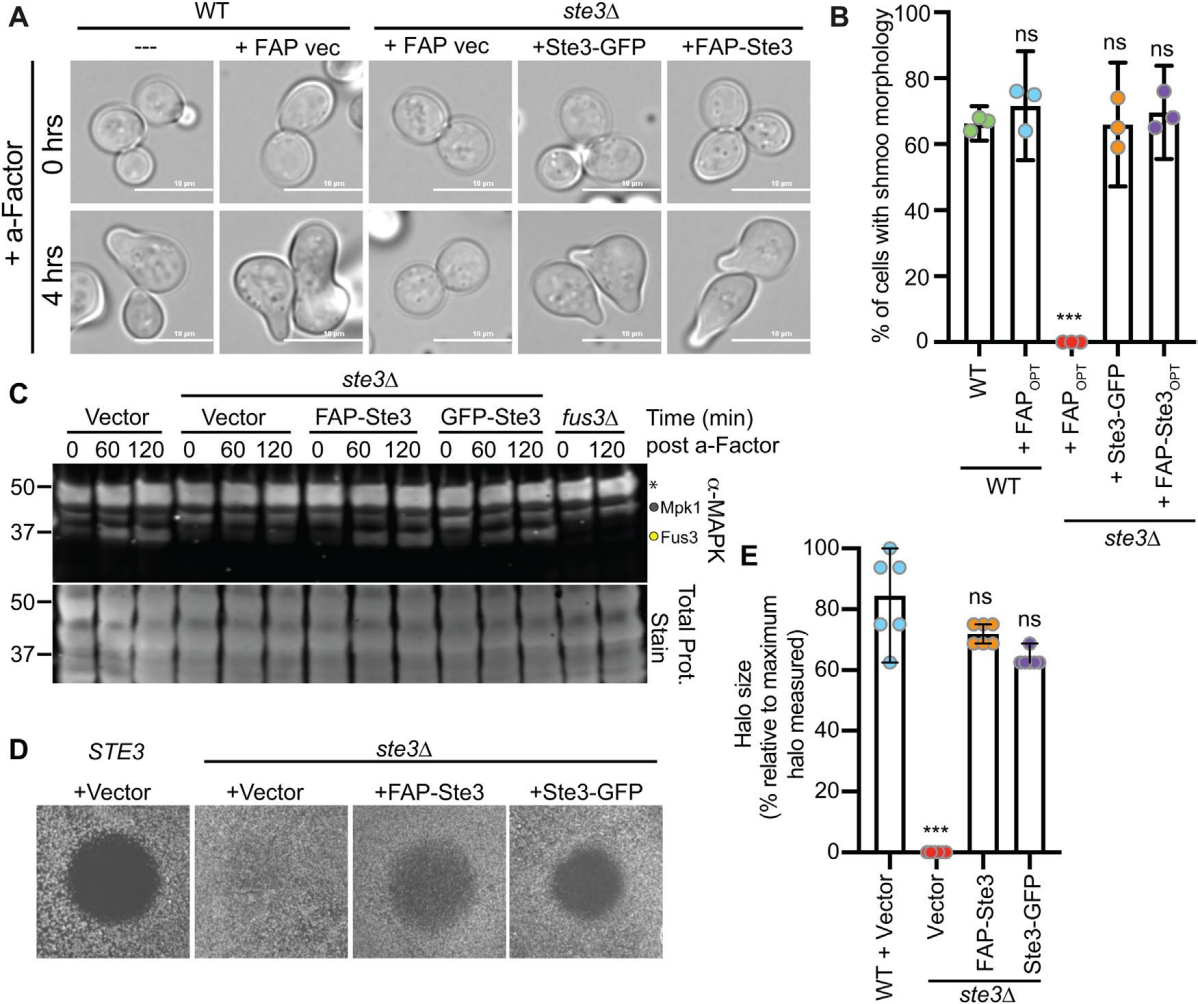
FAP-tagged Ste3 is a functional GPCR in the mating pathway. (A) FAP_optim_, Ste3-GFP, or FAP_optim_-Ste3 were expressed from the *TEF1pr* in WT or *ste3∆* cells and imaged by microscopy before and after a 4-h treatment with 5 μM a-factor. (B) Quantification of the percentage of cells with a shmoo morphology (three biological replicates) from the imaging in panel (A). Mean with 95% confidence interval is shown. Mann-Whitney *t* test was performed comparing each set to WT (not significant = ns; *p* value < 0.001 = ***). (C) Immunoblot of protein extracts from cells treated with 5 μM a-factor for the indicated times. The band indicated with the yellow circle is Fus3, the MAPK activated by the mating pathway; the band immediately above (grey circle) is Mpk1, another MAPK in yeast that is also detected with this antibody; and the top band (asterisks) is an unknown cross-reacting band. (D) Representative a-factor halo assays, in which 5 μg a-factor was spotted at the center of these halos (filter removed for visualization) for either WT or *ste3*∆ cells expressing the indicated plasmids. (E) Quantification of halo diameters as shown in panel D represented as a percentage of the maximal halo size measured across six biological replicates. Student’s *t* test compared experimental values to WT + vector (not significant = ns; *p* < 0.0005 = ***)

We first confirmed that the FAP-Ste3 protein exhibits WT activity, akin to untagged, endogenous Ste3, or the extensively studied Ste3-GFP fusion protein (Urbanowski and Piper, 2001; MacDonald and Piper, 2017; MacDonald *et al.*, 2020). Upon activation of the mitogen-activated protein kinase (MAPK) cascade downstream of Ste3, yeast cells undergo a morphological shift known as “shmoo” formation (Bardwell *et al.*, 1994; Bardwell, 2005), which facilitates the fusion of mating cells. When expressed as the sole copy of Ste3, we found that FAP-Ste3 was as efficient at stimulating shmoo formation as endogenous Ste3 or Ste3-GFP (Figure 6, A and B). In contrast, *ste3*Δ cells expressing FAP_optim_ lacked shmoos (Figure 6, A and B). Next, because the addition of the Ste3 ligand, a-factor, induces expression of the MAPK, Fus3 (Couve and Hirsch, 1996), we assessed Fus3 protein levels. We found that Fus3 increased in a-factor treated cells expressing FAP-Ste3 with similar kinetics to those observed for WT cells with an empty vector or *ste3*Δ cells expressing Ste3-GFP (Figure 6C). Finally, we used halo assays to assess the ability of FAP-Ste3 to induce the hallmark growth arrest associated with activating the mating pathway in response to a-factor. Cells expressing endogenous, untagged Ste3, or *ste3*Δ cells expressing FAP-Ste3 or Ste3-GFP produced halos of equivalent sizes (Figure 6, D and E). Thus, FAP-Ste3 is a functional, a-factor-stimulated GPCR suitable for studies to monitor the mechanism of Ste3 trafficking.

### Deletion of yapsin Mkc7 improves PM detection of FAP-Ste3

**FIGURE 7:**
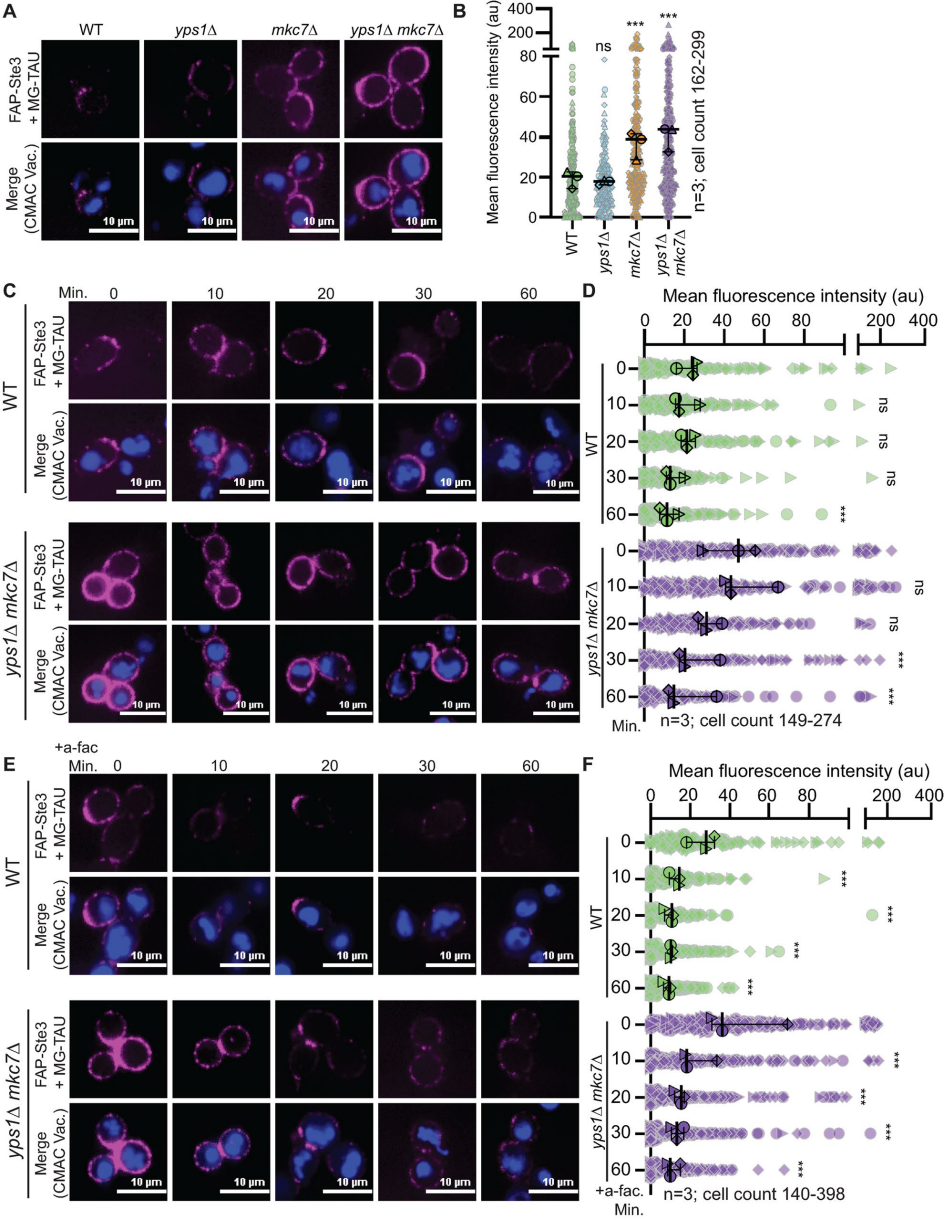
Deletion of yapsin Mkc7 improves FAP-Ste3 fluorescence. (A) Representative confocal microscopy images of FAP_optim_-Ste3 expressed from the *STE3pr* in the genetic backgrounds indicated. Cells were incubated with MG-TAU (impermeant) dye to visualize the surface pool of Ste3 (magenta), and CMAC (blue) stained the vacuoles. (B) Whole-cell fluorescence intensities from cells imaged in panel A were determined using ImageJ for three biological replicates. Data are presented as a Superplot where each cell measured is plotted as a grey outlined shape (circle, triangle, diamond), with the shapes indicating cells from one of the three replicates. The mean fluorescence intensity of each replicate is then plotted as a larger shape outlined in black with the mean for each of the three trials and 95% confidence interval shown with black bars. Kruskal-Wallis statistical analysis with Dunn’s post hoc test was performed to compare the means of the three replicates to the WT control (not significant = ns; *p* < 0.0005 = ***). (C and E) Representative confocal fluorescence microscopy images of FAP_optim_-Ste3 expressed from the *STE3pr* in WT and *yps1∆ mkc7∆* cells either untreated (C) or treated with a-factor (E). Cells were incubated with MG-TAU (impermeant) dye at *t* = 0 to visualize cell surface Ste3 and CMAC-stained vacuoles. The dye was then washed from the cells, and cells were imaged over time (C) or 5 μM a-factor was added, and cells were then imaged over time (E). This allowed us to monitor the steady-state (panel C) or ligand-induced (panel E) turnover of FAP-Ste3 from the PM. (D and F) Whole-cell fluorescence intensity for cells imaged as in panels (C) or (E), respectively, was determined using ImageJ for three biological replicate experiments. The data are presented as a Superplot where each cell measured is plotted as a grey outlined shape, with the shapes corresponding to a single replicate. The mean fluorescence intensity for each replicate is plotted as a circle, triangle, or diamond outlined in black with the mean for each of the three trials and 95% confidence interval shown with black bars. Kruskal-Wallis statistical analysis with Dunn’s post hoc test was performed to compare the means of the three replicates to the WT control (not significant = ns; *p* < 0.0005 = ***).

An earlier study using FAP-tagged Ste2, which resides at the PM of *MATa* cells, found that SCA-dependent fluorescence and receptor stability were improved when the yapsins, which are, glycosylphosphatidylinositol-anchored aspartyl proteases on the cell surface (Krysan *et al.*, 2005) were deleted (Emmerstorfer-Augustin *et al.*, 2018). This increase was likely due to loss of yapsin-dependent cleavage of the FAP tag from Ste2. Consistent with this prior study, we found that deletion of the yapsin Mkc7 significantly increased surface FAP-Ste3 fluorescence in the presence of the cell-impermeant MG-TAU dye (Figure 7, A and B); the absence of another yapsin, Yps1, did not further increase surface fluorescence (Figure 7, A and B). Deleting these aspartyl proteases facilitated the quantitative detection of steady-state and ligand-induced FAP-Ste3 internalization (Figure 7, C–F). Importantly, a-factor induced internalization of FAP-Ste3 was faster (complete after ∼20 min) than the observed constitutive internalization of Ste3, in which internalization continued for ∼60 min (compare Figure 7, C and D to 7, E and F) in the absence of yapsins. We note, however, that Ste3 internalization was challenging to observe in cells when the yapsins were present (i.e., WT cells) because the initial fluorescent signal for FAP-Ste3 was low, and possibly confounded by ongoing FAP cleavage (Figure 7, C–F).

### Assessment of cis-acting sequences on ligand-induced Ste3 endocytosis

**FIGURE 8:**
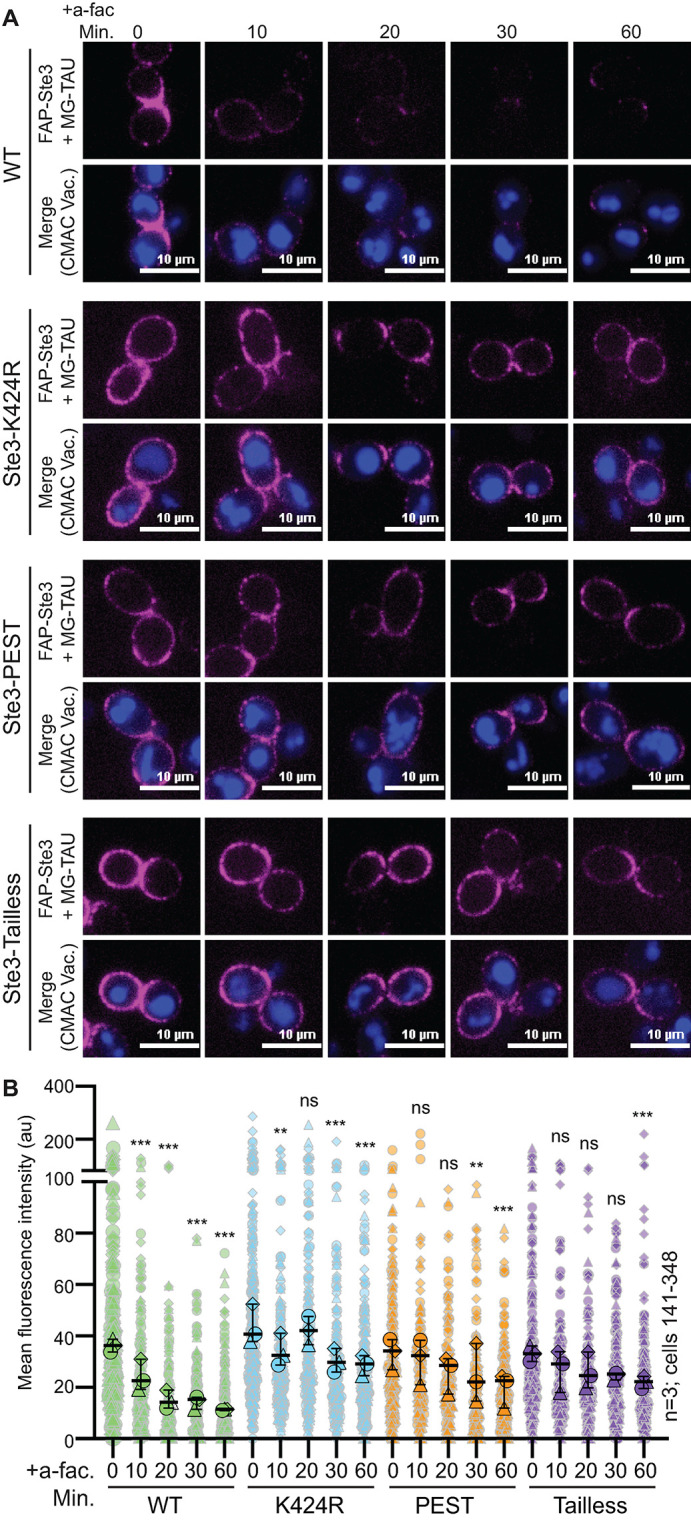
The impact of *cis*-acting sequences on ligand-induced turnover of FAP-Ste3. (A) Representative confocal fluorescence microscopy images of either WT or mutated FAP_optim_-Ste3 expressed from the endogenous *STE3pr* in *yps1∆ mkc7∆* cells treated with 5 μM a-factor for the indicated time. Cells were incubated with MG-TAU (impermeant; magenta) dye at *t* = 0 to visualize cell surface Ste3 and CMAC (blue) stained the vacuoles. The dye was washed from the cells, 5 μM a-factor was added, and cells were imaged over time, allowing ligand-induced turnover of FAP-Ste3 to be monitored. (B) Whole-cell fluorescence intensity for cells imaged in panel A was determined using ImageJ for three biological replicate experiments. The data are presented as a Superplot where each cell measured is plotted as a grey outlined shape, with the shapes corresponding to a single replicate. The mean fluorescence intensity for each replicate is plotted as a larger circle, triangle, or diamond outlined in black with the mean for each of the three trials and 95% confidence interval shown with black bars. Kruskal-Wallis statistical analysis with Dunn’s post hoc test was performed to compare the means of the three replicates to *t* = 0 control for each version of Ste3 (not significant = ns; *p* = < 0.005 = **; *p* < 0.0005 = ***).

The posttranslational modification of the C-terminal tail of many GPCRs regulates endocytosis and trafficking by mediating key interactions with trafficking regulators (Roth and Davis 2000, Zanolari *et al.*, 1992, Dohlman and Thorner 2001). Like other GPCRs, Ste3 has a long C-terminal tail implicated in its endocytosis (Zanolari and Riezman, 1991; Chen and Davis, 2000, 2002). Next, we evaluated the role of Ste3 C-terminal sequences in regulating ligand-induced turnover. Prior studies of these amino acid motifs and their role in Ste3 internalization were primarily limited to biochemical assessment (Roth and Davis, 1996, 2000; Roth *et al.*, 1998), as quantitative assessment of Ste3-GFP endocytosis by live-cell imaging was confounded by high levels of vacuolar Ste3-GFP fluorescence. We generated a FAP-Ste3-Tailless mutant lacking the entire C-terminal tail (amino acids 288-470) and monitored its turnover from the PM relative to the FAP-Ste3 WT control. We found that a-factor-induced turnover of Ste3-Tailless was significantly disrupted, resulting in prolonged receptor retention at the cell surface (Figure 8, A and B). Ste3-Tailless remained at the PM for up to 30 min post a-factor addition, whereas nearly all WT receptors were internalized in this timeframe (Figure 8, A and B).

To further refine our analysis of the Ste3 C-tail, we deleted the PEST-like motif (Ste3-∆PEST; amino acids 413-470), posited to regulate constitutive, but not ligand-induced, Ste3 endocytosis (Roth *et al.*, 1998). While the defect in ligand-induced turnover of Ste3-∆PEST was more modest than that observed for Ste3-Tailless, it was significantly slowed compared to WT Ste3 (Figure 8, A and B). These findings suggest that the PEST sequence not only regulates constitutive turnover but also controls ligand-induced Ste3 internalization.

Finally, we assessed the role of ubiquitination in ligand-induced Ste3 internalization. Selective ubiquitination of membrane proteins at key cytosolic lysine residues is often a critical signal for their endocytosis (Rotin *et al.*, 2000; Herrador *et al.*, 2013), and early studies of Ste3 demonstrated that ubiquitination of the Ste3 C-terminal tail regulates turnover, at least in biochemical assays (Roth and Davis, 1996, 2000; Roth *et al.*, 1998). Though the entire C-tail of Ste3 contains 17 lysines that could serve as putative ubiquitination sites, only three lysines residing within the PEST domain have been shown to alter Ste3 ubiquitination and internalization (Roth and Davis, 1996, 2000; Roth *et al.*, 1998). While past studies indicate that these three lysines (K424, K432, and K453) are somewhat functionally redundant, mutation of K424 to arginine alone significantly delayed Ste3 turnover (Roth and Davis, 2000). Therefore, we made the FAP-tagged Ste3-K424R mutant and assessed endocytic turnover in response to a-factor. Like the PEST deletion, this single point mutation impaired loss of FAP-Ste3 signal at the PM post ligand addition, consistent with reduced endocytic turnover (Figure 8, A and B). Consistent with the importance of K424 ubiquitination, it was difficult to distinguish a difference between Ste3-Tailless, -K424R, or Ste3-∆PEST kinetics (Figure 8, A and B).

### Ligand-Induced endocytosis of Ste3 is regulated by the Aly1, Aly2, and Art1 α-arrestins

**FIGURE 9:**
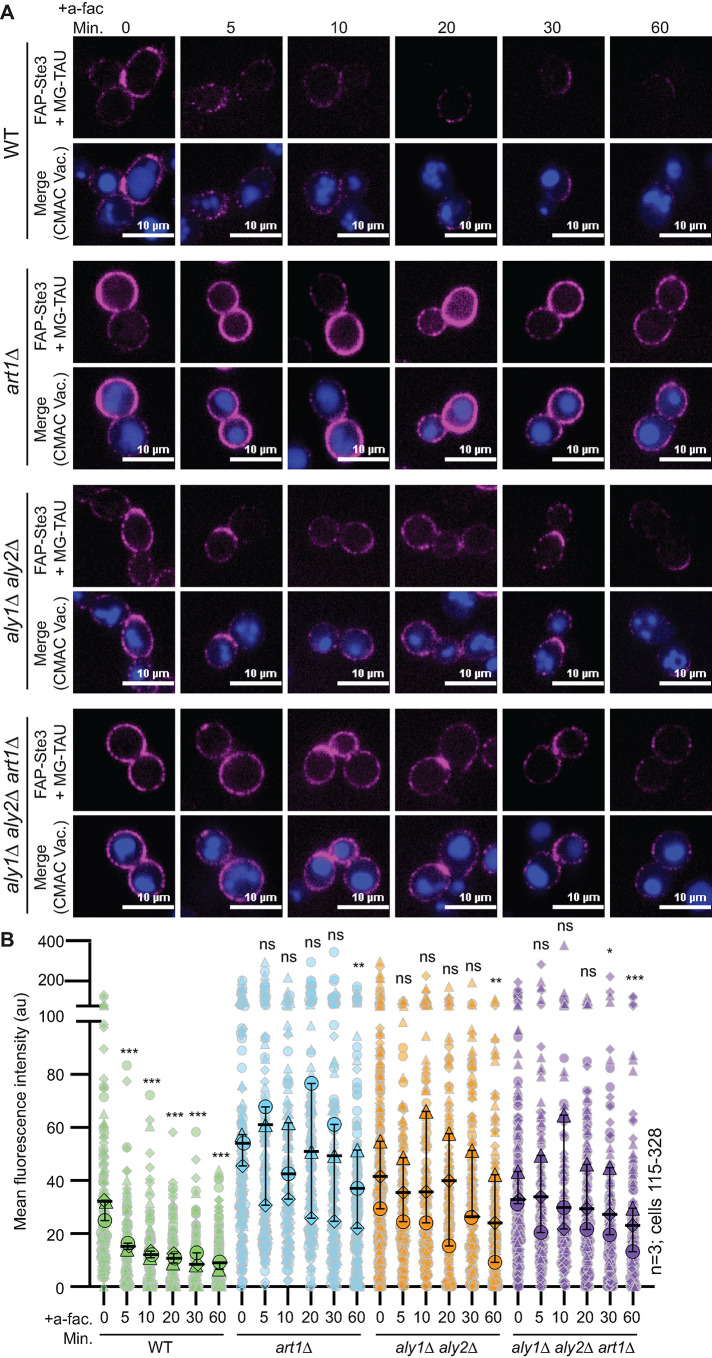
Contribution of α-arrestins to ligand-induced turnover of FAP-Ste3. (A) Representative confocal fluorescence microscopy images of FAP_optim_-Ste3 expressed from the endogenous *STE3pr* in the cells indicated and treated with 5 μM a-factor. Cells were incubated with MG-TAU (impermeant) dye at *t* = 0 to visualize cell surface Ste3, and CMAC stained the vacuoles. The dye was then washed from the cells, 5 μM a-factor was added, and cells were imaged over time. This allows us to monitor the ligand-induced turnover of FAP-Ste3 from the PM. (B) Whole-cell fluorescence intensity for cells imaged in panel A was determined using ImageJ for three biological replicate experiments. The data are presented as a Superplot where each cell measured is plotted as a grey outlined shape, with the shapes corresponding to a single replicate. The mean fluorescence intensity for each replicate is plotted as a circle, triangle, or diamond outlined in black with the mean for each of the three trials and 95% confidence interval shown with black bars. Kruskal-Wallis statistical analysis with Dunn’s post hoc test was performed to compare the means of the three replicates to *t* = 0 control for each of the strains (not significant = ns; *p* = <0.05 = *; *p* = <0.005 = **; *p* < 0.0005 = ***).

**FIGURE 10:**
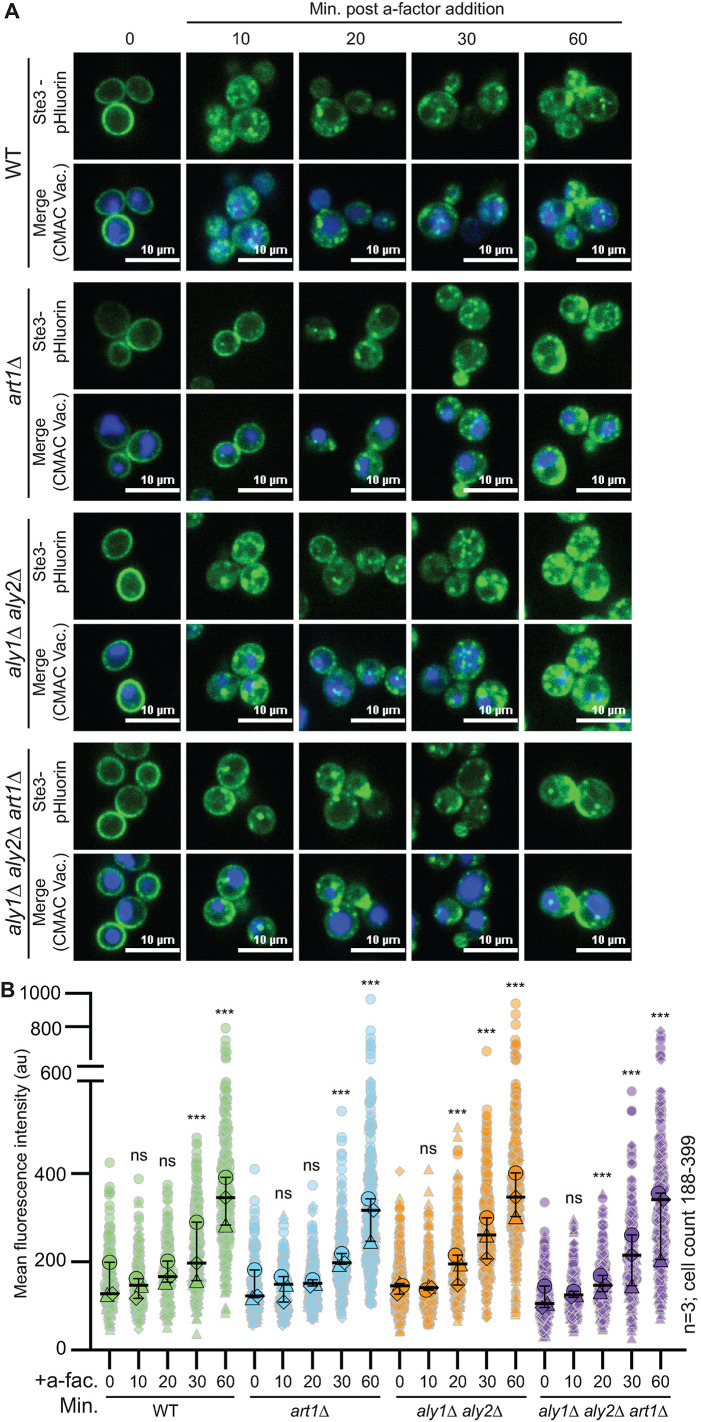
Ligand-induced turnover of Ste3-pHluorin. (A) Representative confocal fluorescence microscopy images are presented of Ste3-pHluorin (green) expressed from the endogenous *STE3* chromosomal locus in either WT or cells lacking the indicated α-arrestin. Cells were either untreated or treated with 5 μM a-factor and CMAC (blue) stained the vacuoles. (B) Whole-cell fluorescence intensity for cells imaged in panel A was determined using ImageJ for three biological replicate experiments. The data are presented as a Superplot where each cell measured is plotted as a grey outlined shape, with the shapes corresponding to a single replicate. The mean fluorescence intensity for each replicate is plotted as a circle, triangle, or diamond outlined in black with the mean for each of the three trials and 95% confidence interval shown with black bars. Kruskal-Wallis statistical analysis with Dunn’s post hoc test was performed to compare the means of the three replicates to *t* = 0 control for each strain (not significant = ns; *p* < 0.0005 = ***).

We previously reported that the constitutive endocytosis of Ste3-pHluorin depends on α-arrestins (Prosser *et al.*, 2015), a class of protein trafficking adaptors conserved from yeast to man (Alvarez, 2008; Lin *et al.*, 2008; Nikko and Pelham, 2009; O’Donnell *et al.*, 2010; O’Donnell and Schmidt, 2019). α-Arrestins serve as a bridge between membrane proteins and the Rsp5 ubiquitin ligase, which ubiquitinates membrane proteins to permit their efficient endocytosis (Lin *et al.*, 2008; Nikko and Pelham, 2009; Becuwe *et al.*, 2012; O’Donnell *et al.*, 2013). In our past work, of the 14 yeast α-arrestins, only Aly1, its paralogue Aly2, and Art1 (aka Art6, Art3, and Ldb19, respectively) stimulated Rsp5-dependent internalization of Ste3 under steady-state conditions (Prosser *et al.*, 2015). Importantly, these findings are supported by analogous studies of FAP-Ste3 (Supplemental Figure S5, A and B). By examining FAP-Ste3 in combination with the MG-TAU dye, we selectively monitored Ste3 abundance at the PM after a-factor addition. As observed when constitutive internalization was assessed (Prosser *et al.*, 2015), cells lacking either Art1, Aly1, and Aly2, or all three of these α-arrestins, exhibited delayed Ste3 internalization after a-factor addition (Figure 9, A and B). Emphasizing the utility of the FAP-tagging system, when we similarly monitored ligand-induced endocytosis using Ste3-pHluorin in WT cells or those lacking α-arrestins, no significant drop in Ste3 fluorescence was observed (Figure 10, A and B; Supplemental Figure S6, A and B).

**FIGURE 11:**
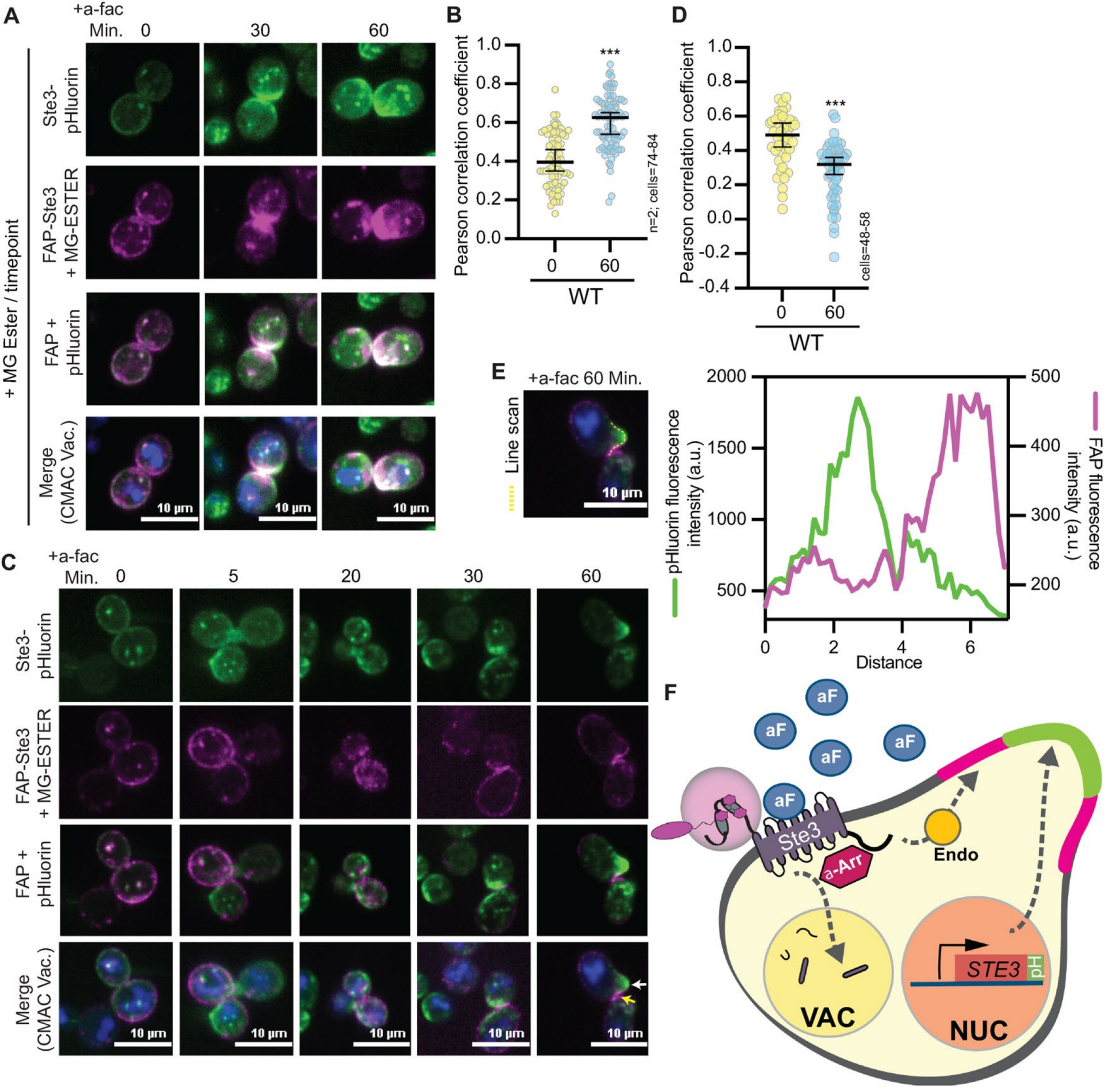
FAP-Ste3 can be used to monitor recycling from the PM. (A) Cells expressing FAP_optim_-Ste3 from a plasmid using the *STE3pr* and chromosomally integrated Ste3-pHluorin were imaged by confocal fluorescence microscopy. Cells were incubated with MG-ESTER and CMAC dye. Once the dye was washed from the cells, 5 μM a-factor was added and the cells were imaged at the indicated timepoints with fresh MG-ESTER dye added before imaging each timepoint. (B and D) Pearson’s correlation coefficient of the FAP-Ste3 and Ste3-pHluorin fluorescent signals in panels A and C, respectively, were determined using the colocalization analysis plugin in Image J. (C) Cells expressing FAP_optim_-Ste3 from a plasmid using the *STE3pr* and chromosomally integrated Ste3-pHluorin were imaged by confocal fluorescence microscopy. Cells were incubated with MG-ESTER and CMAC dye. Once the dye was washed from the cells, 5 μM of a-factor was added, and the cells were imaged at the indicated timepoints with no further dye additions. This allowed us to monitor a single pool of Ste3 at the onset of the experiment with FAP while monitoring the total pool of Ste3 with pHluorin. The yellow arrow points to FAP-Ste3 at the side of the shmoo while the white arrow points to Ste3-pHluorin at the tip of the shmoo. (E) Line scan analysis to monitor the relative distributions of FAP-Ste3 and Ste3-pHluorin at the shmoo tip after pheromone addition. The region used in the line scan is indicated as a dashed yellow line on the image and the fluorescence intensities along this line for GFP (left y-axis) or FAP (right y-axis) are plotted. (F) Model of Ste3 trafficking based on FAP and pHluorin-tagged studies. The addition of Ste3’s ligand, a-factor (aF, blue circle), induces MAPK signaling from the Ste3 receptor and leads to morphology and transcriptional changes in cells that allow for mating. As part of the transcriptional response, *STE3* gene expression is induced, generating new Ste3 protein that is pHluorin tagged. Newly synthesized FAP-Ste3 will not be visualized unless the dye is added at each timepoint (as in Figure 11A). The FAP-Ste3 pool present at the PM before a-factor addition can be visualized selectively using the cell impermeant dye administered just before a-factor treatment (as in Figure 11C). Newly synthesized Ste3-pHluorin localizes to the shmoo tip of cells while “old” FAP-Ste3 that was at the PM before a-factor addition localizes to the sides of the shmoo tip. It is unclear which pathways control these distinct targeting events for Ste3. We find that α-arrestins (Aly1, Aly2, and Art1) are important for the ligand-induced internalization of Ste3 and that the C-tail of Ste3 is needed for its efficient endocytosis. Past work shows that the C-tail is also the site of α-arrestin binding, and the PEST and K424 residues are important cis-regulators of Ste3 internalization. Nuc = nucleus; Vac = vacuole; Endo = endosome.

To ensure that Ste3-pHluorin and FAP-Ste3 behaved similarly, we monitored total FAP-Ste3 post a-factor addition by adding the MG-ESTER dye at every interval during a time course. This contrasts with our prior experiments where we labeled FAP-Ste3 with MG-TAU at the zero timepoint and then monitored the change in distribution for this subset of the receptor (Figure 8, A and B). When MG-ESTER dye is added at each timepoint, we should observe the entire FAP-Ste3 population, which includes the intracellular and cell surface pools of Ste3 before ligand induction and newly transcribed and translated Ste3 after the ligand is added. By adding MG-ESTER dye over time, we found that, like Ste3-pHluorin, FAP-Ste3 also increased in abundance after a-factor addition, and these two differentially tagged receptors colocalized (Figure 11, A and B). Thus, pHluorin- and FAP-tagged Ste3 behave similarly in our assays when the total pool of each protein is monitored.

If we further alter the parameters for the imaging assay, and instead only add MG-ESTER at a single time point before a-factor addition, we can selectively monitor the pool of FAP-Ste3 that is expressed before a-factor addition. In contrast, the Ste3-pHluorin reports on both the preexisting pool of Ste3 and the newly synthesized Ste3 post a-factor addition. In this assay, FAP-Ste3 and Ste3-pHluorin initially colocalized (Figure 11C, timepoints 0, 5, and 10 min and Figure 11D), but after prolonged incubations with a-factor, the preexisting FAP-labeled pool differed relative to the pool of Ste3-pHluorin (Figure 11, C and D; 60-min timepoint). Specifically, by 1 h post a-factor addition, the Ste3-pHluorin pool accumulated at the shmoo tip, whereas FAP-Ste3, which represents the preexisting pool of Ste3 before a-factor addition, partitioned to the side of the shmoo (Figure 11, C and E; Supplemental Figure S6, A and B). These findings suggest differential targeting of the preexisting receptor-present before ligand induction and represented by the FAP-Ste3 population at the sides of the shmoo tip–-versus newly synthesized and exocytosed receptor, which is expressed after a-factor addition and is defined by the Ste3-pHluorin population at the shmoo tip (Figure 11F). In the future, it will be exciting to use these new FAP tools to better define the features that dictate this differential sorting of “old” and “new” Ste3 receptor populations.

## DISCUSSION

### Adaptation of FAP for imaging in yeast

Current approaches to study endocytosis and intracellular trafficking of membrane proteins are limited by their inability to monitor the dynamics of select protein subpopulations. To overcome this challenge, we developed an optimized FAP live-cell imaging system for use in *S. cerevisiae* (Perkins and Bruchez, 2020). To facilitate the use of FAP in yeast imaging studies, we constructed plasmids for building FAP fusions or making FAP-tagged integrations, as well as a suite of FAP-tagged colocalization markers. In our current work, FAP is imaged in the far-red channel (640 excitation/680 emission) when bound to the cell-permeant MG-ESTER dye, allowing for the total pool of protein to be monitored, or the cell-impermeant MG-TAU dye, permitting the surface population of a protein to be analyzed in isolation (Figure 1). These features validate FAP a highly useful probe as few live cell fluorescent tags exploit the far-red channel, which is spectrally distinct from commonly used green, red, and blue fluorophores (i.e., GFP, RFP, BFP, and their derivatives). Importantly, most other approaches also fail to differentially track specific protein subpopulations, as can be achieved with this dual FAP dye system.

To improve the parameters for FAP imaging in yeast, we: i) altered the nucleotide sequence encoding FAP to ensure that the optimal codons for expression and stability in yeast were used, ii) identified the pH range (4.1–7) over which FAP fluorescence is detectable, iii) demonstrated that low pH (<6.0) can induce endocytosis of membrane proteins and should be avoided for trafficking studies in yeast, iv) found that the FAP tag is susceptible to cleavage by vacuolar proteases, which greatly diminishes FAP fluorescence when the protein is sorted to this organelle, and v) confirmed that when FAP is expressed extracellularly it is cleaved by the yapsin aspartyl proteases, demonstrating that, for quantitative endocytic studies, the genes encoding yapsins should be deleted. With these parameters defined, we used FAP technology to study ligand-induced endocytosis of the Ste3 GPCR.

### Ligand-induced Ste3 endocytosis and recycling

The function of Ste3 and its downstream signaling pathway in controlling yeast mating have been actively investigated for decades (Bardwell *et al.*, 1994; Bardwell, 2005). Initially, biochemical studies defined the machinery that mediates Ste3 endocytosis and postendocytic recycling of Ste3 to the PM (Roth and Davis, 1996, 2000; Roth *et al.*, 1998; Chen and Davis, 2000, 2002). Importantly, many cell surface receptors in mammals analogously recycle to the PM after internalization, and some are dually targeted for postendocytic recycling to the PM and degradation in the lysosome (Tomas *et al.*, 2014). While the endocytosis of many membrane proteins is ubiquitin-dependent, postendocytic sorting to the vacuole is far better described than the endosomal recycling pathways, which are still being characterized (Bonifacino and Weissman, 1998; Sorkin and Von Zastrow, 2002). For Ste2, the counterpart of Ste3 in *MATa* cells, endocytosis leads to vacuolar targeting and degradation (Schandel and Jenness, 1994; Urbanowski and Piper, 2001; MacDonald and Piper, 2017; MacDonald *et al.*, 2020). However, protease-shaving assays measuring Ste3 PM abundance after a-factor treatment suggested that internalized Ste3 is recycled to the PM (Chen and Davis, 2000). Ste3 recycling has been confirmed by image analysis and used to help define proteins needed for postendocytic recycling to the PM (MacDonald and Piper, 2017; Laidlaw *et al.*, 2022, a, b). In this study, we sought to further assess cis- and *trans*-acting factors in the endocytosis and recycling of Ste3 using live cell imaging with FAP-tagged Ste3.

We showed that FAP-tagged Ste3 was a functional receptor and could activate the mating pathway in *MATα* cells similar to WT Ste3 or Ste3-GFP (Figure 6). Past elegant biochemical approaches defined the Ste3 residues required for its efficient endocytosis post ligand addition (Roth and Davis, 1996, 2000; Roth *et al.*, 1998; Chen and Davis, 2000, 2002). However, these experiments relied on cell fixation and cellular fractionation and thus did not monitor cell surface populations of Ste3 in real-time. The FAP imaging technique enabled us to map Ste3 trafficking dynamics with increased resolution to provide more detailed spatial information. We found that the bulk of Ste3 internalization occurs within the first 10 to 20 min following exposure to a-factor, rather than on the longer timeframes (40–120 min) reported previously (Chen and Davis, 2000). Interestingly, we noted that FAP-Ste3 was not evenly distributed at the PM but had a somewhat punctate distribution in the membrane. This is similar to the punctate patterning described for many PM proteins, which can partition into discrete PM subdomains with unique protein and lipid compositions (Lingwood and Simons, 2010; Schuberth and Wedlich-Soldner, 2015; Sezgin *et al.*, 2017). It will be interesting in future studies to see which, if any, subdomain Ste3 may partition to at the cell surface.

Consistent with earlier findings, we demonstrated that the Ste3 C-terminal tail was essential for endocytosis (Roth and Davis, 1996, 2000; Roth *et al.*, 1998). Within the tail, the PEST region was posited to be required only for steady-state receptor turnover (Roth *et al.*, 1998; Roth and Davis, 2000). However, we found that disruption of sequences within the PEST motif similarly blocks ligand-induced endocytosis of Ste3. Three key lysines in the PEST motif are ubiquitinated to permit Ste3 endocytosis (Roth and Davis, 1996, 2000). We found that mutation of just one of these lysines, K424, was sufficient to disrupt ligand-induced Ste3 endocytosis, causing PM retention of a significant receptor population even after 60 min of ligand exposure. Past studies showed that Ste3 ubiquitination depends on Rsp5 (Rotin *et al.*, 2000; Chen and Davis, 2002; Abazari *et al.*, 2015; Prosser *et al.*, 2015), yet Ste3 lacks Rsp5 interaction motifs. Indeed, we previously identified α-arrestins as important regulators of Rsp5-dependent Ste3 basal internalization via clathrin-mediated endocytosis (Prosser *et al.*, 2015).

When comparing the trafficking of FAP- and pHluorin-tagged Ste3, we found that the Ste3 synthesized in response to a-factor accumulates at the shmoo tip about 1 h after a-factor exposure (Figure 11F). Previous work detailing the transcriptional responses to a-factor did not elaborate on the localization of newly synthesized receptors (Hagen and Sprague, 1984). This increase in Ste3 abundance due to a-factor-induced transcriptional activation of Ste3 obscures endocytic dynamics when using fluorescent tags such as pHluorin that label the complete pool of Ste3 in cells. In contrast, the ability to selectively visualize distinct spatial and temporal pools of Ste3 using the FAP tag, in combination with the cell-impermeant dye, highlights the power of the imaging technology reported here. Using differentially tagged Ste3 populations, we showed that Ste3 present in the cell before a-factor addition and the newly synthesized Ste3 are targeted to spatially distinct PM locations (Figure 11F). Our data suggest a sorting mechanism that distinguishes “old” from “new” Ste3 and directs the receptor based on its past location in the cell.

What features dictate Ste3 postendocytic sorting? Ste3 has been used as a model recycling cargo thanks to work that helped dissect factors needed for early endosomal recycling in yeast (MacDonald and Piper, 2017). These analyses were performed under basal, but not ligand-induced conditions, and identified Rcy1 (an F-box protein involved in endosomal recycling), Ist1 (a protein needed for endosomal recycling), and Nhx1 (a sodium/potassium exchanger required for vacuole fusion) as endosomal factors that facilitate Ste3 recycling to the PM (MacDonald and Piper, 2017; Laidlaw *et al.*, 2022a). Another study employing a deubiquitinase (DUb) fusion to fluorescently tagged Ste3 suggested that Gpa1, the Gα subunit of the mating pathway, and components of the glucose sensing machinery (e.g., the glucose sensing GPCR, Gpr1; a regulatory subunit of the Glc7 phosphatases, Reg1; and two transcriptional repressors, Mig1and Mig2) were also necessary for efficient recycling of Ste3 to the PM (Laidlaw *et al.*, 2022b). In future studies, we will explore the role of these recycling factors with FAP-Ste3 and Ste3-pHluorin to evaluate their impact on Ste3 endocytosis and recycling in response to a-factor, thereby providing a real-time analysis of their contributions.

### α-Arrestins operate in both basal and ligand-induced GPCR endocytosis

In addition to defining the role of key *cis*-acting sequences in ligand-induced Ste3 trafficking that reside at the “tail”, we identify the α-arrestins required for PM turnover of Ste3. Consistent with our early studies of constitutive turnover of Ste3 (Prosser *et al.*, 2015), we find that three α-arrestins, Aly1, Aly2, and Art1, are needed for ligand-induced Ste3 internalization. Here, we find that both constitutive and ligand-induced endocytosis of Ste3 is impaired in the absence of Art1, Aly1, and Aly2 (Figure 11F). However, these α-arrestins do not appear to impact Ste3 equivalently. In cells lacking Art1, Ste3 retention at the PM post a-factor treatment is robust and the receptor is evenly distributed across the cell surface (Figures 9; Supplemental Figure S6). In contrast, in cells lacking Aly1/Aly2, Ste3 PM retention is less striking and shorter-lived (Figure 9). In addition, concentrated patches of Ste3 arise in *aly1*∆ *aly2*∆ cells, suggesting either that the Aly adaptors cannot internalize Ste3 from a subdomain of the PM or that Ste3 recycles to specific PM regions more effectively in the absence of these α-arrestins, resulting in patches of receptor close to bud neck or shmoo sites. These findings raise the possibility of functional partitioning for α-arrestins, which might allow them to regulate the trafficking of discrete pools of PM or intracellular receptors. More generally, numerous cargos are internalized by α-arrestins, yet mechanisms underlying the functional redundancy for these trafficking adaptors remain poorly understood.

The role of α-arrestins in regulating mammalian GPCRs remains somewhat controversial (Shea *et al.*, 2012; Aubry and Klein, 2013; Han *et al.*, 2013). However, their role in regulating GPCRs in yeast is established (Alvaro *et al.*, 2014; Prosser *et al.*, 2015; Emmerstorfer-Augustin *et al.*, 2018). It would be surprising if the mammalian α-arrestins could not similarly regulate GPCRs, especially in light of the high-content studies that have identified many β-arrestin-independent GPCRs (Moo *et al.*, 2021). Notably, mammalian α-arrestins have been shown to engage GPCRs, but for the two best-described examples to date, the α-arrestins are not operating in controlling endocytosis of these GPCRs. More specifically, mammalian α-arrestin ARRDC3 interacts with the β2-adrenergic receptor at endosomes in a ligand-independent manner to control the intracellular recycling of this receptor (Tian *et al.*, 2016) but can also help drive ubiquitination of protease-activated receptor 1 (PAR1) to stimulate PAR1’s lysosomal degradation (Dores *et al.*, 2015). These findings support the idea that human α-arrestins are likely able to engage GPCRs, though the precise sequences needed for α-arrestin-GPCR interaction have not been mapped.

In yeast, specific sequences in the Ste3 C-terminal tail are needed for its endocytosis. The defects in ligand-dependent endocytosis we observed for Ste3 C-tail mutants could be due to the elimination of posttranslational regulatory sites or disruption of the binding sites for endocytic regulators, such as the α-arrestins and/or clathrin-binding adaptor proteins. The amino acids needed for α-arrestin-GPCR association have also not yet been mapped. Considering that three α-arrestins mediate the internalization of Ste2 and Ste3 (i.e., Rod1, Rog3, and Art1 or Aly1, Aly2, and Art1, respectively), there is an opportunity for both a common regulatory sequence (i.e., for Art1) and divergent regulatory sequences (i.e., for Rod1/Rog3 vs. Aly1/Aly2) in Ste2 and Ste3. To date, only a few α-arrestin interaction interfaces have been defined for any membrane protein, but based on the few examples, it appears that α-arrestins preferentially associate with acidic patches on cargo proteins (Lin *et al.*, 2008; Wawrzycka *et al.*, 2019; Ivashov *et al.*, 2020; Barata-Antunes *et al.*, 2022). Our earlier work demonstrated that α-arrestins can bind to the C-tails of Ste3 and Ste2, suggesting that key interactions are needed to recruit α-arrestins in these regions (Alvaro *et al.*, 2014; Prosser *et al.*, 2015). With FAP technology, we are well-positioned to define how α-arrestins recognize GPCRs, filling a significant knowledge gap in the field and further establishing α-arrestins as bona fide regulators of GPCR trafficking and signaling.

## MATERIALS AND METHODS

### Yeast strains and growth conditions

Yeast strains are listed in Supplemental Table 1 and derived from the BY4742 (*MATα*) genetic background of *S. cerevisiae* (S288C in origin)*.* Deletion strains were built using a PCR-based method described previously (Longtine *et al.*, 1998). Yeast cells were grown at 30°C and cultured in synthetic complete (SC) medium (2% glucose, yeast nitrogen base without amino acids, supplemented with amino acid drop-out mixtures for selection) or yeast extract peptone dextrose medium (YPD; Johnston *et al.*, 1977). Liquid medium was filter sterilized, and for plate medium, 2% wt/vol agar was added before autoclaving. Yeast cultures were grown overnight, then reinoculated (A_600_ = 0.2 or 0.3) and grown to midexponential log phase (∼4 h to reach an A_600_ = 0.8*–*1.0) before experimentation.

### Plasmids and DNA manipulations

Plasmids used in this work are listed in Supplemental Table 2. Plasmid constructs were built using PCR amplification with Phusion High Fidelity DNA polymerase (Thermo Fisher Scientific, Waltham, MA) and sequence validated through Sanger sequencing (Genewiz, South Plainfield, NJ). Plasmid maps were generated using SnapGene software (Insightful Science, Chicago, IL). Gene Blocks of the original and optimized sequence of FAP were obtained from GeneWiz (GeneWiz, South Plainfield, NJ). Codon optimization was done using the JCat; Technical University of Braunschweig, Brunswick, Germany; Grote *et al.*, 2005). JCat employs a CAI to generate an optimized DNA sequence by measuring the codon usage bias, which is the frequency of synonymous codon occurrence and cognate tRNAs usage in an organism (Grote *et al.*, 2005). The FAP-tagging plasmids and those expressing these FAP-tagged cellular markers are all available on Addgene (see Supplemental Table 2 or Addgene global deposit number 84326). Plasmids were transformed into yeast cells using the lithium acetate method (Ausubel, 1991) and selected using SC media lacking specific amino acids or YPD medium supplemented with antibiotics.

### BSA coated tubes

The a-factor peptide is hydrophobic and tends to adhere to glass surfaces during purification. To ensure a-factor remained in solution, all tubes for a-factor treatments were coated with 1% wt/vol bovine serum albumin (BSA; Sigma-Aldrich, Waltham, MA) solution. Tubes were coated by filling them with the BSA solution and incubating at room temperature with rotation overnight. Following incubation, the BSA solution was removed, and tubes were rinsed with sterile water before use.

### Shmoo morphology assessment

To evaluate yeast morphology before and after a-factor treatment, cells were grown to saturation in SC medium at pH 6.6, inoculated in fresh medium (A_600_ = 0.3), and grown to mid-exponential log phase (∼A_600_ = 0.8). Equal densities of cells were collected by centrifugation and resuspended in 1 ml of fresh SC medium. Cells were plated onto concanavalin A-coated (MP Biomedicals, Solon OH, USA) 35-mm glass bottom microwell dishes (MatTek Corp., Ashland, MA) and the zero time-point was imaged using differential interference contrast (DIC) microscopy on a Nikon Ti inverted microscope (Nikon Instruments, Tokyo, Japan). Cells were transferred into BSA-coated glass culture tubes, 5 μM of a-factor mating pheromone (Zymo Research; 1 mg/ml working stock) was added, and cells were incubated at 30°C for 4 h before imaging and assessing shmoo formation.

The morphological composition of the cell populations was assessed using FIJI 2.0.0, a version of ImageJ (NIH, Bethesda, MA). Cells were assigned into “yeast-form” or “shmoo” populations using the cell counter tool. The percentage of cells with shmoo morphology was calculated, and statistical significance was determined using a Student’s *t* test in Prism Software, version 10 (GraphPad, Boston, MA).

### FAP Staining and Confocal Fluorescence Microscopy

To determine the localization and abundance of FAP-tagged Ste3, cells were grown as described above. Equal densities of cells (∼A_600_ 0.8) were collected by centrifugation and resuspended in 98 μl of fresh SC medium. A final concentration of 10 μM Cell Tracker Blue CMAC (7-amino-4-chloromethylcoumarin) dye (Life Technologies; Carlsbad, CA) and 1 μM of MG-TAU dye (cell permeant dye αRed-np1, Spectra Genetics, Pittsburgh, PA; 1:100 dilution of stock) or 1 μM of MG-ESTER (cell permeant dye αRed-p1, Spectra Genetics; 1:200 dilution of stock) was added to visualize vacuoles or the FAP tag at the PM or in whole cells, respectively. Cells with dye were incubated at 30°C with agitation for 15 min. Following incubation, cells were washed twice in 1 ml of fresh medium and transferred to a new BSA-coated tube. The zero timepoint was imaged from these cells, then 5 μM a-factor (Zymo Research, Orange, CA) was added to the rest of the cells, and they were incubated at 30°C with shaking, and sampled and imaged at the indicated timepoints. To image cells, 75 μl of cell suspension with 75 μl of fresh medium were plated onto 35-mm glass bottom microwell dishes (MatTek Corp., Ashland, MA) coated with concanavalin A (MP Biomedicals, Solon OH, USA). It is important to note that while MG itself can be toxic to yeast cells, the MG-derived dyes are not; even for cells incubated in these MG-derived dyes for prolonged periods (i.e., ∼20 h), there is no inhibition of yeast cell growth, unlike the robust inhibition of yeast cell growth that occurs when the same concentration of MG is used to treat cells (Szent-Gyorgyi *et al*., 2008).

For pHluorin imaging, cells were grown overnight to saturation in SC media, inoculated in fresh medium (A_600_ = 0.2) and grown to midexponential log phase (∼A_600_ = 0.8). Equal densities of cells were collected by centrifugation and resuspended in fresh SC medium, to which 10 μM CMAC dye was added to visualize vacuoles and cells were visualized post a-factor addition as indicated above. Cells were similarly incubated with MG-ESTER or MG-TAU dyes where simultaneous pHluorin and FAP-tagged Ste3 imaging was performed.

All confocal fluorescence microscopy was performed on a Nikon Eclipse Ti inverted microscope (Nikon Instruments, Tokyo, Japan) outfitted with a swept field confocal scan head (Prairie Instruments, Middleton, WI), an Apo 100X objective (NA 1.49), an Agilent monolith laser launch (Agilent Technologies, Santa Clara, CA), and an Andor iXon3 camera (Oxford Instruments, Andor Technologies, Belfast, Northern Ireland). All images within an experiment were captured using identical parameters as controlled by the NIS-Elements software (Nikon Instruments). Images for figures were adjusted evenly within a figure panel, unless otherwise indicated, using NIS-Elements.

### Flow cytometry measurements

To quantitatively assess FAP fluorescence in dynamic populations, yeast cells containing the indicated FAP-tagged marker were cultured to midlogue phase (as indicated above) and fluorescence was analyzed using an Attune Nxt Flow Cytometer (Thermo Fisher Scientific). Flow rate was set to collect 12.5 μl/min or 100,0000 events. The RL2 laser was used for excitation of the FAP probe and detected using the 720/730 filter. A threshold of 25 × 1000 was applied to the forward scatter to capture yeast cells. Data was analyzed using FlowJo software (Becton, Dickinson & Company, Franklin Lakes, NJ) to gate samples in forward and side scatter to include only single yeast cell events when measuring fluorescent values.

To define the pH sensitivity of the FAP-fluorescence, cells were grown in pH 6.6 medium overnight, inoculated into fresh pH 6.6 medium (A_600_ = 0.2) and grown for 2 h at 30°C. Cells were then collected by centrifugation, washed, and shifted to media of differing pH (adjusted to 4.1, 4.6, 5.1, 5.8, or 6.6 using hydrochloric acid or sodium hydroxide) and grown for 2 h in these new pH conditions. Equal densities of cells (∼A_600_ = 0.8) were collected by centrifugation and resuspended in 99 μl of fresh media of the corresponding pH for that experiment and 1 μM of either MG-TAU or MG-ESTER dye was added. Cells were incubated for 15 min at 30°C with agitation, washed twice, and resuspended in 1 ml of fresh media and placed in a new tube. Fluorescence was measured using an Attune NxT Flow Cytometer (Thermo Fisher Scientific). The voltage settings for acquisition were 215 for FSC, 325 for SSC, and 450 for RL2. Cells that were not stained with the FAP dye were used to gate nonfluorescent cells.

### Quantitative imaging analyses and statistical tests

All images were manually quantified using FIJI software version 2.0.0 to measure fluorescence intensities. A 2-pixel wide line was hand-drawn around the perimeter of the PM to establish the ROI. The ROI was overlayed on the 405 channel to validate the vacuole costaining (CMAC). Mean pixel intensity of the background was subtracted from each ROI as described (O’Donnell *et al.*, 2015). At least three full imaging fields and 75 cells were analyzed for each dataset. Though hundreds to thousands of cells were analyzed for an individual experiment, at least three biological replicate experiments were performed for each condition. These data were collated into a Superplot (Prism Software, GraphPad; Lord *et al.*, 2020). The resulting data were evaluated using Prism software, where statistics were performed on the mean values from the three replicate experiments and not on the full population distributions. Kruskal-Wallis statistical analyses with Dunn’s post hoc test were used to define significant changes, denoted by *p value <0.1, **p value <0.01, ***p value <0.001, and ns *p* value >0.1.

To assess vacuolar fluorescence, the CMAC vacuolar stain was converted to a mask using FIJI software (as described in O’Donnell *et al*., 2015) that defined the vacuole regions of interest. This mask was then applied to the FAP channel (as for Figure 3G) and the median FAP signal in the vacuole measured. On occasion, perivacuolar compartments were included in this fluorescence measure as the CMAC mask was not always precise enough to differentiate between the vacuole and perivacuolar (likely MVBs) compartments, which may be docked onto the vacuole surface. Mean pixel intensity of the background was subtracted from each ROI as described (O’Donnell *et al.*, 2015). Statistical analyses were performed using a Student’s *t* test for Figure 3G.

To define the colocalization of FAP-tagged Ste3 with pHluorin-tagged Ste3 (Figure 11, B and D), Pearson’s Correlation Coefficient was determined using the colocalizations plugin in FIJI software (Image J, National Institutes of Health [NIH]). To demonstrate the spatial distribution change between Ste3-pHluorin and FAP-Ste3, line scans were performed using the plot profile analysis tool in FIJI software (Image J, NIH) and plotting the values in Prism (GraphPad).

### Yeast protein extraction and immunoblot analysis

To analyze protein abundance, yeast whole-cell protein extracts were made by growing cells in SC medium with appropriate nutrient selection to midexponential phase at 30°C (A_600_ of 0.7–0.8) and then harvesting equivalent densities of cells by centrifugation. Cell pellets were flash-frozen in liquid nitrogen and stored at –80°C. To make extracts, pelleted cells were lysed, and proteins precipitated using the trichloroacetic acid extraction method as described (Volland *et al.*, 1994). Protein precipitates were solubilized in sodium dodecyl sulfate [SDS]/urea sample buffer (40 mM Tris [pH 6.8], 0.1 mM EDTA, 5% SDS, 8 M urea, and 1% β-mercaptoethanol; O’Donnell *et al.*, 2013) and heated to 37°C for 15 min. Extracts were resolved by SDS–PAGE, and proteins were identified by immunoblotting. Either Revert 700 Total Protein stain (LI-COR BioSciences, Lincoln, NE) of the membranes or anti-Zwf1 antibody (MilliporeSigma, St. Louis, MO) was used as a loading and transfer control. For immunoblotting, primary antibodies against Myc (catalogue# MA1980, Thermo Scientific, Waltham, MA), RFP (catalogue# 600-401-379, Rockland Immunochemicals, Pottstown, PA), ERK1/2 (C-9, sc 514302; Santa Cruz Biotechnology, Santa Cruz, CA), or Zwf1 (catalogue# A9521, MilliporeSigma, St. Louis, MO) were employed at the dilutions indicated from the manufacturers. Antimouse or antirabbit secondary antibodies conjugated to IRDye-800 or IRDye-680 (LI-COR BioSciences) were detected using the Odyssey CLx infrared imaging system (LI-COR BioSciences).

## Supplementary Material



## Abbreviations used:

CFTR: cystic fibrosis transmembrane conductance regulator
ER: endoplasmic reticulum
FAP: fluorogen activating protein
FP: fluorescent proteins
GFP: green fluorescent protein
GPCR: G-protein coupled receptor
MAPK: Mitogen activated protein kinase
MG: malachite green
MG-Ester: cell permeant MG-derived dye
MG-B-Tau: cell impermeant MG-derived dye
MVB: multi-vesicular body
PM: plasma membrane
SCA: single-chain antibody
WT: wild-type.
